# A machine-learning-based approach to predict early hallmarks of progressive hearing loss

**DOI:** 10.1016/j.heares.2025.109328

**Published:** 2025-06-06

**Authors:** Federico Ceriani, Joshua Giles, Neil J Ingham, Jing-Yi Jeng, Morag A Lewis, Karen P Steel, Mahnaz Arvaneh, Walter Marcotti

**Affiliations:** 1 School of Biosciences, University of Sheffield, Sheffield, S10 2TN, UK; 2 Department of Automatic Control and Systems Engineering, University of Sheffield, Sheffield, S1 4DT, UK; 3 Centre for Machine Intelligence, University of Sheffield, Sheffield, S10 2TN, UK7; 4 Wellcome Sanger Institute, Hinxton, UK; 5 Wolfson Sensory, Pain and Regeneration Centre, Guy’s Campus, King’s College London, London SE1 1UL, UK; 6 Neuroscience Institute, University of Sheffield, Sheffield S10 2TN, UK

**Keywords:** age-related hearing loss, auditory brainstem responses, machine learning, diagnosis.

## Abstract

Machine learning (ML) techniques are increasingly being used to improve disease diagnosis and treatment. However, the application of these computational approaches to the early diagnosis of age-related hearing loss (ARHL), the most common sensory deficit in adults, remains underexplored. Here, we demonstrate the potential of ML for identifying early signs of ARHL in adult mice. We used auditory brainstem responses (ABRs), which are non-invasive electrophysiological recordings that can be performed in both mice and humans, as a readout of hearing function. We recorded ABRs from C57BL/6N mice (6N), which develop early-onset ARHL due to a hypomorphic allele of *Cadherin23* (*Cdh23*^*ahl*^), and from co-isogenic C57BL/6NTac^*Cdh23+*^ mice (6N-Repaired), which do not harbour the *Cdh23*^*ahl*^ allele and maintain good hearing until later in life. We evaluated several ML classifiers across different metrics for their ability to distinguish between the two mouse strains based on ABRs. Remarkably, the models accurately identified mice carrying the *Cdh23*^*ahl*^ allele even in the absence of obvious signs of hearing loss at 1 month of age, surpassing the classification accuracy of human experts. Feature importance analysis using Shapley values indicated that subtle differences in ABR wave 1 were critical for distinguishing between the two genotypes. This superior performance underscores the potential of ML approaches in detecting subtle phenotypic differences that may elude manual classification. Additionally, we successfully trained regression models capable of predicting ARHL progression rate at older ages from ABRs recorded in younger mice. We propose that ML approaches are suitable for the early diagnosis of ARHL and could potentially improve the success of future treatments in humans by predicting the progression of hearing dysfunction.

## 1 Introduction

Progressive hearing loss results in a decrease in hearing sensitivity and ability to understand speech. Among the different forms of progressive hearing loss, age-related hearing loss (ARHL) is the most common sensory deficit in humans, affecting communication and leading to social isolation, depression and diminishing cognitive abilities (Gates & Mills, 2005; Livingston *et al*. 2024). Currently, there are no treatments to prevent or cure ARHL (Wang & Puel, 2020). ARHL is a heterogeneous dysfunction, which results from the cumulative effects of ageing on the auditory system, such as cellular senescence, as well as additional intrinsic (e.g. genetic predisposition, Ingham *et al*. 2019) and extrinsic (e.g. environmental noise) factors. Because of this complex aetiology, the progression of the disease varies between individuals, resulting in different severity and degree of progression of hearing loss. Hearing function in clinical and pre-clinical settings can be examined through a non-invasive electrophysiological test based on the auditory brainstem response (ABR). However, the effects of hearing loss, other than an obvious increase in auditory thresholds, are often difficult to detect using ABR tests. Thus, ARHL is normally diagnosed only after patients start losing key hearing abilities, such as being unable to distinguish words in noisy conditions. This is usually an indication that some severe or irreversible damage has already happened to the sensory cells or neurons that send sound information to the brain. Therefore, as we develop therapies to target ARHL, such as gene-based replacement interventions or small molecules (Lv *et al*. 2024; Schilder *et al*. 2024), there is also a pressing need to improve the diagnostic tools to detect and predict the progression of the dysfunction at an early stage. As with any medical condition, treating a disease in its early stages increases the likelihood of successful treatment.

Machine learning (ML) techniques are increasingly being explored as tools to improve disease diagnosis and treatment (Goecks *et al*. 2020; Sidney-Gibbons & Sidney-Gibbons, 2019). These techniques leverage advanced algorithms to analyse large datasets, uncovering patterns that may be elusive even to well-trained experts. By identifying complex features in high-dimensional clinical data that correlate strongly with patient phenotypes, ML algorithms can be developed to predict the presence of a disease (Banerjee *et al*. 2023). In the auditory field, significant progress is being made in applying ML to hearing healthcare and research (Chen *et al*. 2021; Shew *et al*. 2019, Cha *et al*. 2019, Crowson *et al*. 2023, Chen *et al*. 2024), and there is a growing emphasis on the leveraging of ML-based digital tools to automate hearing assessment (Wasmann *et al*. 2022). However, the potential of these computational techniques to develop diagnostic tools for the early detection of progressive forms of hearing loss remains largely unexplored.

Here, we applied ML to ABR data with the goal of detecting early signs of ARHL in mice and forecasting its progression. We recorded ABRs from the commonly used C57BL/6N (6N) mouse strain and from the co-isogenic strain C57BL/6NTac^*Cdh23+*^ (6N-Repaired, Mianné et al. 2016) at 1, 3, 6, 9 and 12 months of age. The 6N mice carry a hypomorphic allele in the Cadherin 23 gene (*Cdh23*^*ahl*^, Johnson et al. 1997; Noben-Trauth et al. 2003), which leads to progressive early-onset hearing loss starting from about 3-6 months of age. Similar to ARHL in humans (Gates & Mills, 2005), the progression of hearing loss in 6N mice begins at the higher frequencies and worsens over time, resulting in profound hearing loss by 15 months of age (Jeng et al. 2020a; 2020b; Jeng et al. 2021). In contrast, the co-isogenic 6N-Repaired strain, which are corrected for the *Cdh23*^*ahl*^ mutation using CRISPR/Cas9 (Mianné et al. 2016), maintains better hearing than 6N mice into old age, especially for tone sensitivity for frequencies of 12 kHz and above (Mianné et al. 2016; Jeng et al. 2020b). We trained ML models through supervised learning using longitudinal ABR data as input features and genotype (i.e., mouse strain, 6N or 6N-Repaired) as target outputs. We demonstrate that, by recognising anomalies in the ABRs, the ML models were able to detect the mice with the *Cdh23*^*ahl*^ allele in the very early stages of ARHL. This approach was validated on unseen data of two independently acquired datasets, demonstrating the broad validity and generalisability of our conclusions. Finally, we used ML to forecast the future progression of the hearing capabilities of young adult mice up to 1 year of age. This work highlights the benefit of using ML for the early diagnosis of ARHL, providing a foundation for future studies exploring its applicability to human datasets.

## 2 Methods

### 2.1 Ethical Statement

The animal work was licensed by the UK Home Office under the Animals (Scientific Procedures) Act 1986 (Sheffield: PCC8E5E93 and PP1481074; King’s College London: P053FFC4C) and was approved by the relevant Ethical Review Committees (University of Sheffield: 180626_Mar). Mice had unlimited access to food and water. For the *in vivo* recording of auditory brainstem responses (ABRs), mice were anaesthetised using intraperitoneal injection of ketamine (100 mg/Kg body weight, Fort Dodge Animal Health, Fort Dodge, USA) and xylazine (10 mg/Kg, Rompun 2%, Bayer HealthCare LLC, NY, USA). At the end of the *in vivo* recordings, mice were either culled by cervical dislocation or recovered from anaesthesia with intraperitoneal injection of atipamezole (1 mg/Kg). Mice under recovery from anaesthesia were returned to their cage, placed on a thermal mat and monitored over the following 2-5 hrs.

### 2.2 Auditory brainstem responses

Two independent datasets of auditory brainstem responses (ABRs) from different mouse cohorts were used in this study. ABRs from the **primary cohort** were collected at the University of Sheffield from 104 female mice (50 6N and 54 6N-Repaired mice). For all the mice, ABR recordings were performed at 1 month of age, and for some, recordings were also performed at 3, 6, 9 and 12 months of age. These mice were born over a period of 5 months and were housed in the same room within the animal facilities at the University of Sheffield, thus experiencing similar levels of noise exposure throughout the duration of the study. ABRs from the **replication cohort** were collected at King’s College London from both males and females at 1 month of age (85 6N and 103 6N-Repaired mice).

Following the onset of anaesthesia (see *Ethics statement* above) and the loss of the retraction reflex with a toe pinch, mice were placed onto a heat mat (37°C) in a soundproof chamber (MAC-3 acoustic chamber, IAC Acoustic, UK). Subdermal electrodes were placed under the skin behind the pinna of each ear (reference and ground electrode) and on the vertex of the mouse (active electrode) as previously described (Ingham *et al*. 2019; Ingham *et al*. 2011). Sound stimuli were delivered to the ear by calibrated loudspeakers (MF1-S, Multi Field Speaker, Tucker-Davis Technologies, USA) placed directly in front of the mouse 10 cm (Sheffield) or 20 cm (King’s College London) from the nose. Sound pressure was calibrated with a low-noise microphone probe system (ER10B+, Etymotic, USA). Experiments were performed using a customised software (Ingham *et al*. 2011) driving an RZ6 auditory processor (Tucker-Davis Technologies). Auditory thresholds were estimated from the resulting ABR waveform and defined as the lowest sound pressure level (measured in decibel, dB SPL) where any recognisable feature of the waveform was visible. Responses were measured for clicks (which cover a broad range of frequencies, 0.01 ms duration) and pure tones at frequencies of 3, 6, 12, 18, 24, 30, 36 and 42 kHz (5 ms duration, 1 ms rise/fall time). Stimulus sound pressure levels were typically 0-95 dB SPL, presented in steps of 5 dB SPL. The brainstem response signal was averaged over 256 repetitions. Tone bursts were 5 ms in duration with a 1 ms on/off ramp time, which was presented at a rate of 42.6/sec. The order of sound stimulus presentation was consistent for all ABR recordings. Responses to click stimuli were recorded first (0 to 95 dB), followed by pure tones from 15 dB to 95 dB at 3 kHz and 6 kHz. Finally, stimuli at varying frequency from high (42 kHz) to low (12 kHz) were presented. This process was repeated in 5 dB increments from 15 dB to 95 dB.

Wave 1 amplitudes and latencies were measured using a semiautomatic approach using custom software (doi:10.5281/zenodo.12606227). Automatic identification was manually reviewed and, if required, adjusted to the correct peak. Wave 1 amplitude was calculated as the difference between the amplitude of the first peak and the first trough of the ABR waveform; the latency was calculated as the delay of the Wave 1 peak from the beginning of the recording.

To evaluate the models on a dataset different from the one it was trained on, we found that an alignment procedure was required to maximise model accuracies. This alignment required the shifting of the ABR waveforms from the replication cohort to the left by 0.55 ms. This was likely due to the different distances between mouse and the speaker (~10 cm, accounting for 0.29 ms time difference) and variations in hardware (e.g., differences in electrical delays and timing of sound delivery). The alignment was achieved by removing 54 timepoints at the beginning of the replication cohort trace and at the end of the primary cohort trace (to maintain the same number of features between the two datasets). This procedure, which did not alter the shape and time course of the ABR waveforms, was sufficient to align waveform peaks between the two cohorts. The parameters for feature shifting were determined from ABRs of 6N-Repaired mice from the training datasets from the two cohorts. Note that this transformation, while ensuring correspondence of the features in the two datasets, does not eliminate latency differences between the two strains, as both are shifted by the same amount.

### 2.3 Implementation and evaluation of machine learning models

We developed ML models to address two different tasks: 1) identifying which mice carried the *Cdh23*^*ahl*^ mutation from their ABRs (classification task) and 2) forecasting the future progression of hearing function in mice (regression task). For the classification task, we used six different classifiers as the basis of our models: Random Convolutional Kernel Transform (ROCKET), Hierarchical Vote Collective of Transformation-based Ensembles V2 (HIVE COTE V2.0), Extreme Gradient Boosting (XGBoost), Random Forest (RF), multilayer perceptron (MLP) and Support Vector Machine (SVM) classifier.

The ROCKET algorithm (Dempster *et al*. 2020) classifies time series by applying numerous random filters to the data, extracting key values from the resulting feature maps, and using these values to train a simple, efficient model. This approach is both fast and accurate, transforming complex time series into an easy-to-handle format for effective classification. HIVE COTE V2.0 is a heterogeneous meta ensemble for time series classification (Middlehurst *et al*. 2021) that builds on the ROCKET. This algorithm is more computationally expensive but has shown very high levels of accuracy when utilised to classify other time series data. XGBoost (Extreme Gradient Boosting, Chen *et al*. 2016) and Random Forest are ensemble models that use decision trees as base learners which are widely used due to their accuracy and interpretability. They have proven successful in the classification of electrophysiological data (Edla *et al*. 2018) for a range of applications. Along with these machine learning algorithms the MLP classifier was selected as a simple neural network option to explore. Finally, we selected the SVM classifier as a good base classifier which has been proven to work effectively with minimal computational cost in a wide range of applications. For the regression task, we trained random forest regressor models to predict the outcome of three continuous parameters at different ages (see below). Some of the hyperparameters were tuned using 5-fold grid search cross validation applied to the training set, optimising for F1 score (classification task) or the negative mean squared error (regression task). The hyperparameters of the models are summarised in S6 Table.

**Fig 1:**
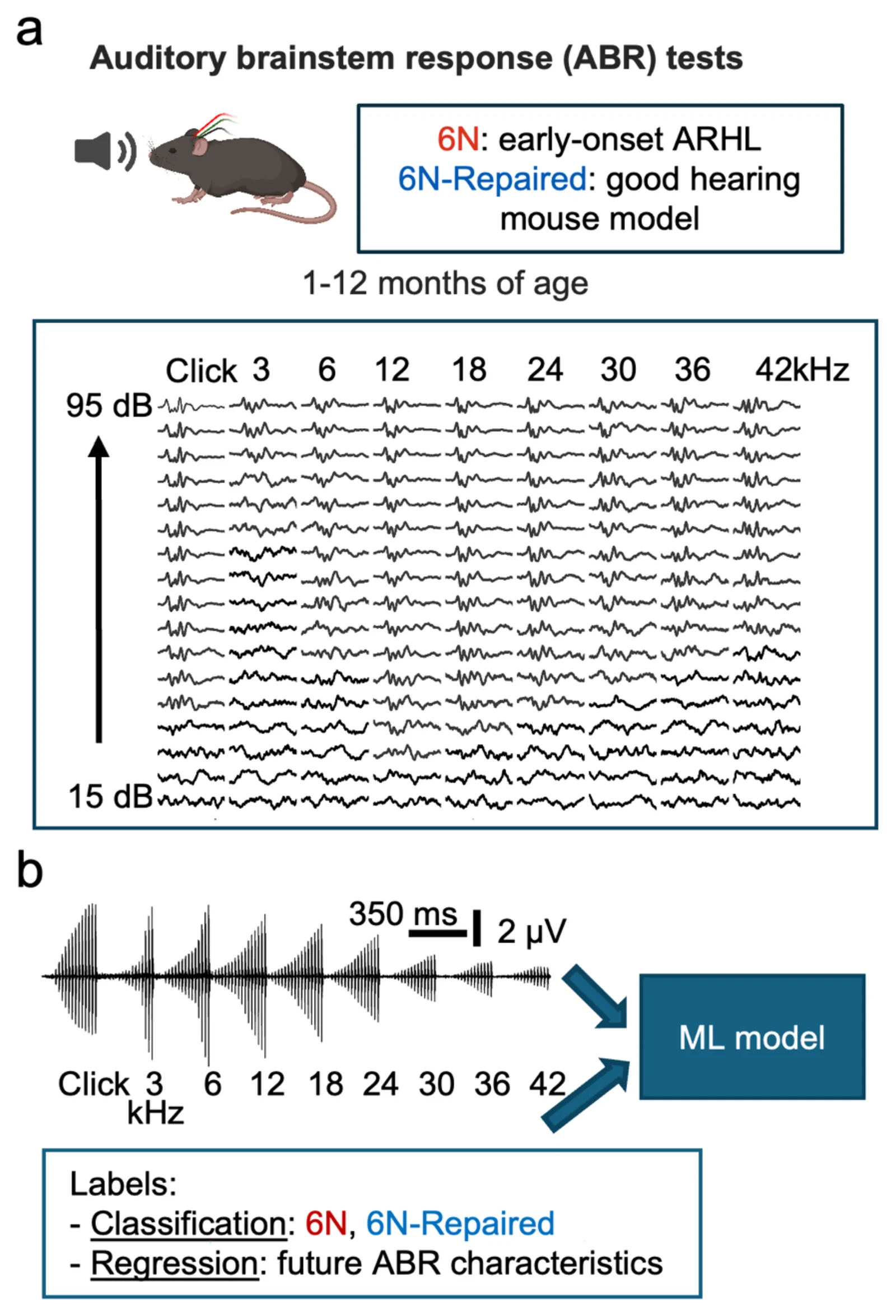
Schematic representation of the work. (**a**) Auditory brainstem responses (ABRs) were recorded from a cohort of 1-month-old 6N (50 mice) and 6N-Repaired mice (54 mice). Some mice were also tested at 3, 6, 9 and 12 months of age. Anaesthetised animals were presented with auditory stimuli comprising clicks and pure tones of frequencies ranging from 3 to 42 kHz and stimulus intensities ranging from 15 dB SPL to 95 dB SPL in 5 dB SPL increments. Bottom panel shows an example of an ABR recording from a 6N mouse. Each of the traces in the matrix lasts 10 ms and represents a response to one combination of stimulus intensity and click/tone frequency. (**b**) Responses to individual intensities/frequencies combinations were concatenated in a single trace and used as input features for the machine learning (ML) models.

Models were implemented using the *scikit-learn* (Random Forest classifier and regressor, SVM, Multi-layer perceptron, Pedregosa *et al*. 2011), *xgboost* (XGBoost, Chen *et al*. 2016) and *sktime* (HIVE COTE V2.0, ROCKET, Löning *et al*. 2019) python packages. Different ABR waveforms resulting from stimulation with individual intensities/frequencies combinations were concatenated in a single univariate trace and used as input features for the ML models (Fig 1b, Fig 3a, Fig 5a, Fig 8d).

For the classification task, ML algorithms were trained through supervised learning using the concatenated ABR waveforms of 1-month old animals as input features and the genotype (6N vs 6N-Repaired) as labels. In all analyses, the “6N” class (i.e., “mice with early-onset ARHL”) was treated as the positive class. All classifiers were preceded by an ANOVA F-test feature selection step, which retained 10% of the features (i.e., ABR timepoints). In this step, the F-statistics scores were calculated between the two classes for every input feature and ranked, and only the 10% top scoring features were preserved as model inputs. By reducing the dimensionality of the dataset, focusing on the most relevant predictors, this method is effective in improving training time and reducing overfitting.

We trained and tested the models on data from two laboratories (primary and replication cohorts) either separately or combined. In every instance, the datasets were randomly split into train and test data, with the training data containing 75% of the mice and the test the remaining 25%. For tasks which involved evaluating the models on data of different laboratories, the whole primary and replication cohort datasets were used either for training or testing (see Results).

We provided two separate evaluations of the models. First, in order to ensure models were working correctly, we performed repeated 5-fold cross validation on the training set collecting 4 different metrics:

- recall (also called sensitivity, or true positive rate), defined as $\frac{\;\text{True}\;\text{positives}\;}{\;\text{True}\;\text{positives}\;\text{+}\;\text{False}\;\text{negatives}\;}$, i.e. the ability of a classifier to correctly identify the positive class (6N);
- specificity (or true negative rate), defined as $\frac{\;\text{True}\;\text{negatives}\;\;}{\;\text{True}\;\text{negatives}\;\text{+}\;\text{False}\;\text{positives}\;}$, i.e. the ability of a classifier to correctly identify the negative class (6N-Repaired);
- precision, defined as $\frac{\;\text{True}\;\text{positives}\;}{\;\text{True}\;\text{positives}\;\text{+}\;\text{False}\;\text{positives}\;}$, a measure of the accuracy of positive predictions;
- receiver operating characteristic area under the curve (ROC AUC), i.e. the area under the false positive rate (1-specificity) vs. recall curve, which offers a threshold-independent measure of a model’s performance.

For all classifiers, the threshold for distinguishing between the two classes was set to a probability of 50%. Models were then trained on the whole training set and confusion matrices were calculated on the test set. We opted to include both cross validation and test set assessment as the relatively small size of the test set limits our ability to draw strong conclusions from its performance alone. However, it allows for comparison with manual classification on the same set of mice.

For the regression task, ML algorithms were trained through supervised learning using the concatenated click ABR waveforms recorded at 1 month and 3 months of age as input features and three different parameters measured at 6, 9 or 12 months of age (see Results) as targets. For wave 1 latency prediction, values for waveforms below the auditory threshold were imputed using the highest latency measured for click stimuli for each mouse. For one mouse for which no detectable ABR signal was present at 12 months of age, missing latency values were imputed using the highest latency for click stimuli observed across all other mice in the dataset. For wave 1 amplitude prediction, values for waveforms below the auditory thresholds were set to zero. Similarly to the classification task, regression models were preceded by a feature selection step based on univariate linear regression tests, which return F-statistics and p-values, and only the 10% top scoring features were preserved as model inputs. The datasets were randomly split into train and test data, with the training data containing 75% of the mice and the test the remaining 25%. Performances of regression models were evaluated as mean absolute error (MAE) averaged across the results of a repeated 5-fold cross validation step on the training set (5 repeats). Moreover, the coefficient of determination (*R*^*2*^) and MAE were calculated on predictions made on the test set.

The Shapley Additive explanations (SHAP) method implemented in the *shap* python module was used for Shapley value estimation (Lundberg & Lee, 2017). The TreeSHAP method was used to estimate Shapley values for tree-based models (Random forest and XGBoost), while KernelSHAP was used for the SVM model. Shapley values were calculated on the test set, using the training set as the background distribution. Feature importances were calculated by averaging the absolute Shapley values computed across all train instances. Feature importances were smoothed with a Savitzky-Golay filter with polynomial order equal to 1 and window size of 0.42 ms (“Global” models) and 0.22 ms (“Click” models) for visualisation purposes.

Machine learning model implementation, data analysis and figure plotting were conducted using python (version 3.11.8) primarily utilising the scikit-learn (version 1.4.1) and sktime (0.27.0) modules. Computations were performed on a MacBook Pro with M1 processor and 16 GB of RAM (MacOS 15.0, kernel Darwin 24.0.0), and on a workstation equipped with an Intel Xeon Silver 4210R CPU and 256 GB of RAM (Windows 11 Pro for workstations).

### 2.4 Comparison between ML and manual classification

Three human annotators were asked to blindly label the ABR dataset for comparison with the ML models. Each annotator reviewed ABR data for all samples in both the training and test sets and categorized each instance according to the mouse strain. Annotators were presented with randomised ABR stacks containing responses to click and pure tones (Fig 3) or clicks alone (Fig 5). Individual ABR waveforms were shown to human experimenters without additional overlays or statistical summaries. Annotators assessed auditory thresholds as part of the classification. Each of the three annotators was an expert in mouse ABR recording and analysis, with specific knowledge of the progressive high frequency hearing loss phenotype of 6N mice compared to 6N-Repaired mice. Predictions on the test set ABRs alone were directly compared to those of the six “Global” and “Click” models (Fig 3f, Fig 5f). For the “Global” dataset, average predictions (±SD) of the three annotators on the whole (train and test) set were as follows: recall 66.7% ± 13.6%; specificity 82.7% ± 18.9%; precision 81.9% ± 14.1%. Average predictions for the “Click” dataset on the whole (train and test) set were: recall 44.7% ± 8.3%; specificity 67.9% ± 7.4%; precision 56.4% ± 1.7%. The results from the three annotators were subsequently evaluated for inter-rater reliability using Fleiss’ Kappa, a statistical measure of agreement among multiple raters.

### 2.5 Statistical analysis

Statistical analysis of experimental data was conducted using either the Aligned Rank Transform (ART) ANOVA, followed by Wilcoxon rank-sum tests with Holm-Bonferroni correction for pairwise post-hoc comparisons, or standard ANOVA with Tukey’s Honestly Significant Difference post-hoc test. For model performance comparisons on the primary cohort, cross validation metrics were evaluated using the Friedman test followed by the Nemenyi post-hoc test. To compare model performances across different datasets (primary cohort/replication cohort/combined or “Global”/”Click”) and model types, we used mixed-effects linear models with model type and dataset as fixed effects and the cross-validation splits as random effects to account for non-independence within repeated measures. A significance level of P < 0.05 was used to determine statistical significance.

## 3 Results

To demonstrate the possibility of ML to detect early signs of a progressive form of deafness, we first acquired ABRs using standardised protocols from a cohort of 1-month-old 6N (*n* = 50) and 6N-Repaired (*n* = 54) mice (**primary cohort**, Fig 1a). To avoid the impact of sex variability in the progression and severity of age-related hearing loss (Nolan, 2020), only female mice were included in the primary cohort. A subset of these mice was aged up to 1 year and ABRs were recorded at regular intervals (3, 6, 9 and 12 months). This approach allowed us to test the efficacy of ML models in learning to distinguish between the two mouse strains based solely on ABRs of 1-month-old mice (classification task). Moreover, we tested whether it was possible to predict the progression and degree of hearing loss at older ages based on ABR data from young adult mice (regression task). To achieve this, we trained several ML algorithms through supervised learning using: 1) ABR data of 1-month old animals as input features and the genotype as labels (classification task) and 2) ABR data recorded at 1 and 3 months of age as input features and ABR characteristics at older ages as target (regression task, Fig 1b).

### 3.1 Auditory thresholds of 6N and 6N-Repaired mice between 1 and 12 months of age

**Fig 2:**
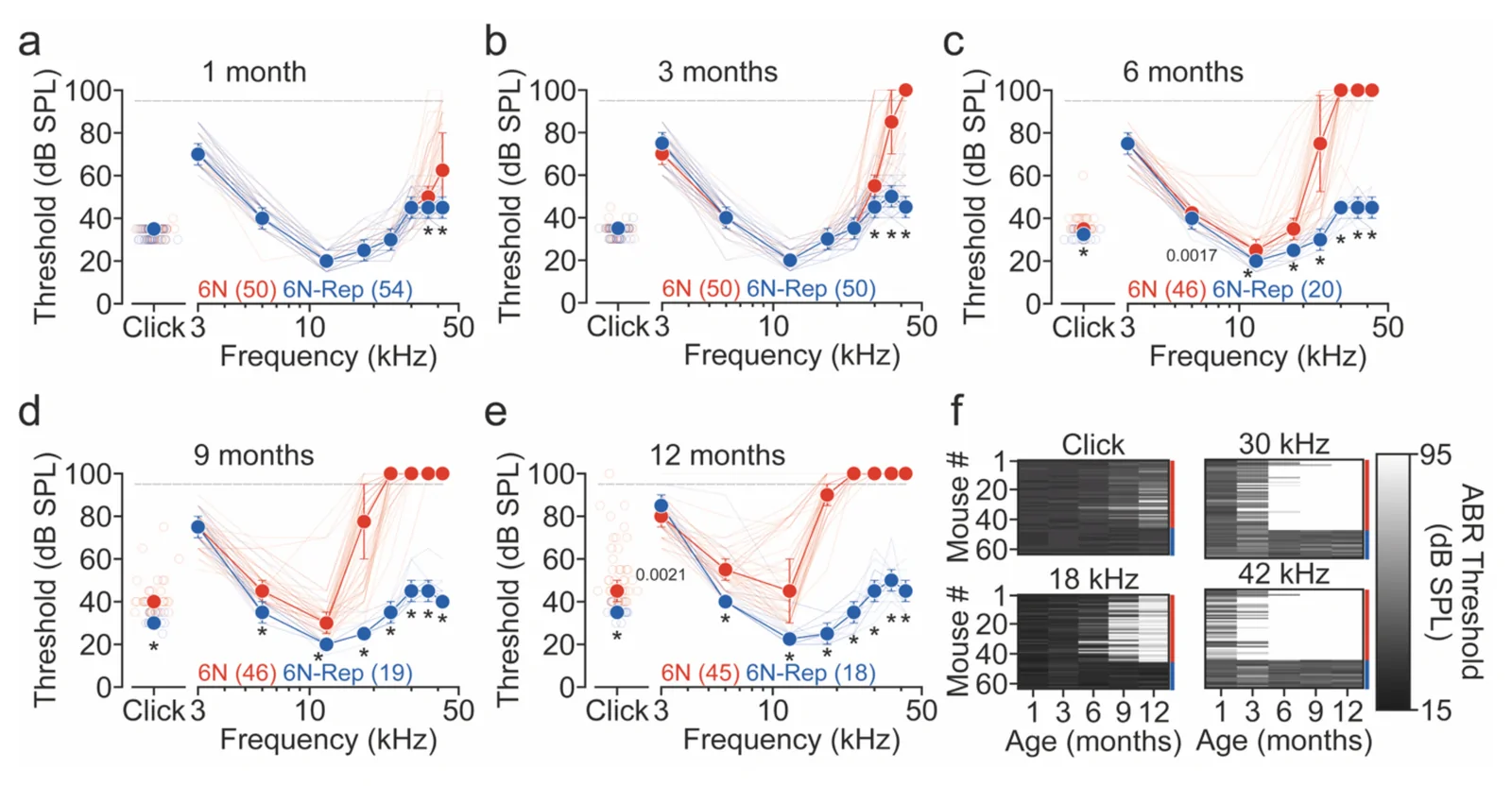
Age-dependent change in ABR thresholds in 6N and 6N-Repaired mice. (**a-e**) ABR thresholds for click and pure tone stimuli at different ages. Solid points represent median ± median absolute deviation, while individual lines represent audiograms of individual mice. The number of mice for each genotype is indicated in parenthesis. The same mice cohort was repeatedly tested at different ages. Note that not all mice were investigated at all age timepoints. Significant differences between the two genotypes are indicated next to the data points (*: P < 0.0001, pairwise Wilcoxon rank-sum test, ART ANOVA). At 1 month of age, auditory thresholds were already significantly different between the two strains (P < 0.0001, ART ANOVA). (**f**) Rasterplots showing auditory thresholds as a function of age for four different stimuli (click, 18, 30, 42 kHz) in the 63 mice (45 6N mice and 18 6N-Repaired mice) that were evaluated at all ages tested. Note the progressive increase in auditory thresholds of 6N mice (red band) compared to 6N-Repaired mice (blue band).

We first determined the progression of hearing loss of our primary cohort of 50 6N and 54 6N-Repaired female mice (Fig 2). We found that at 1 month of age both mouse strains showed similar ABR thresholds (Fig 2a) and waveforms across most sound stimuli (S1 Fig), except for a small increase in the median threshold of 6N mice at the two highest frequencies tested (36 kHz and 42 kHz, P < 0.0001, pairwise Wilcoxon rank-sum test, ART ANOVA, Fig 2a). Difference between the audiograms of the two strains became progressively more evident at older ages (Fig 2b-e, S2 Fig). At 3 months, several 6N mice had undetectable ABRs at all intensities for stimuli of 36 and 42 kHz (36 kHz: 19 6N mice out of 50; 42 kHz: 29 mice out of 50) and significantly raised threshold at 30 kHz compared with 6N-Repaired mice (Fig 2b). Between 6 and 12 months, ABR thresholds were significantly different between the two strains for all stimuli except the lowest frequency tested (3 kHz) at 6 and 9 months (Fig 2c-e). As previously shown (Jeng *et al*. 2020), we found that the progression of hearing loss was variable in 6N mice (Fig 2f), with threshold differences between individual mice of up to 75 dB from 6 months onwards (Fig 2c-e).

### 3.2 Using ML models to predict the presence of the *Cdh23^ahl^* allele from ABRs of young mice

**Fig 3:**
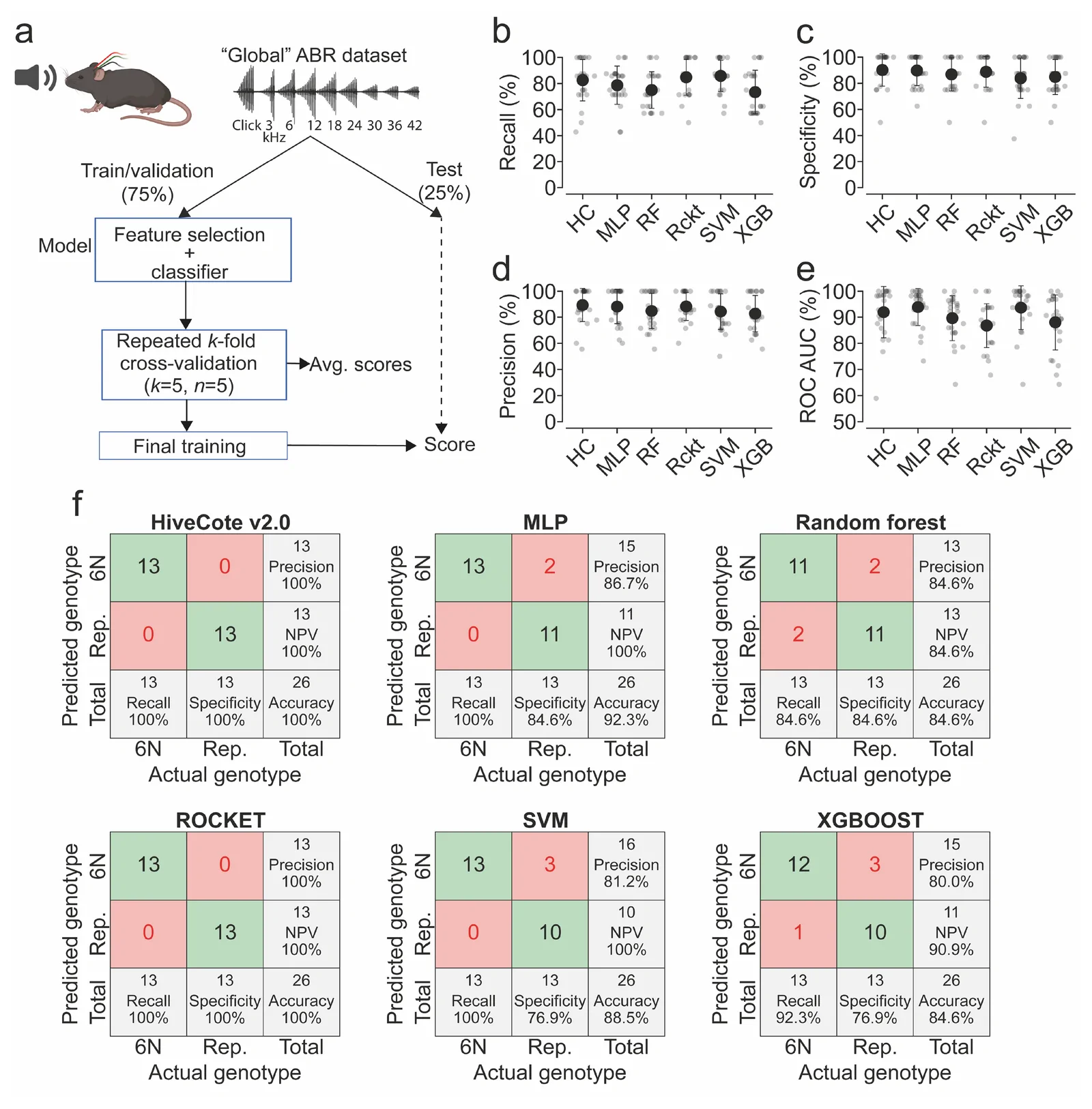
ML models can accurately predict the presence of the *Cdh23*^*ahl*^ allele from ABR waveforms early on. (**a**) Data flow of the ML training/ testing process. The ABR dataset from 1-month old mice in the primary cohort was randomly split into a train/validation set (75% of mice) and a test (hold-out) set (25% of mice). The same train/test split was consistently used across all the models. The models consisted of an ANOVA feature selection step, where the 10% top scoring features (timepoints) were selected (see Methods), followed by one of six classifiers. Models were initially evaluated using repeated stratified *k*-fold cross-validation (*k* = 5 splits and 5 repeats). The 25 scores produced by this step were averaged to provide a measure of the overall performances of the models. Finally, the models were trained on the entire training/validation set from the primary cohort and final scores were obtained by testing predictions on the held-out test set. (**b-e**) Average metrics of the six models trained on the whole ABR (click and 8 tones) as estimated in the 5×5 cross-validation step. Solid dots represent the mean ± SD. Smaller dots indicate the scores from individual folds in the cross-validation step (25 scores per model, see also S1 Table and S2 Table for statistical comparisons). (**f**) Confusion matrices highlighting the performances of the models trained on the whole ABR (click and 8 tones) on the final test set. HC: HiveCoteV2.0; MLP: multilayer perceptron. RF: random forest; Rckt: ROCKET; SVM: support vector classifier; XGB: XGBoost. Rep: 6N-Repaired. NPV: negative predictive value.

**Fig 4:**
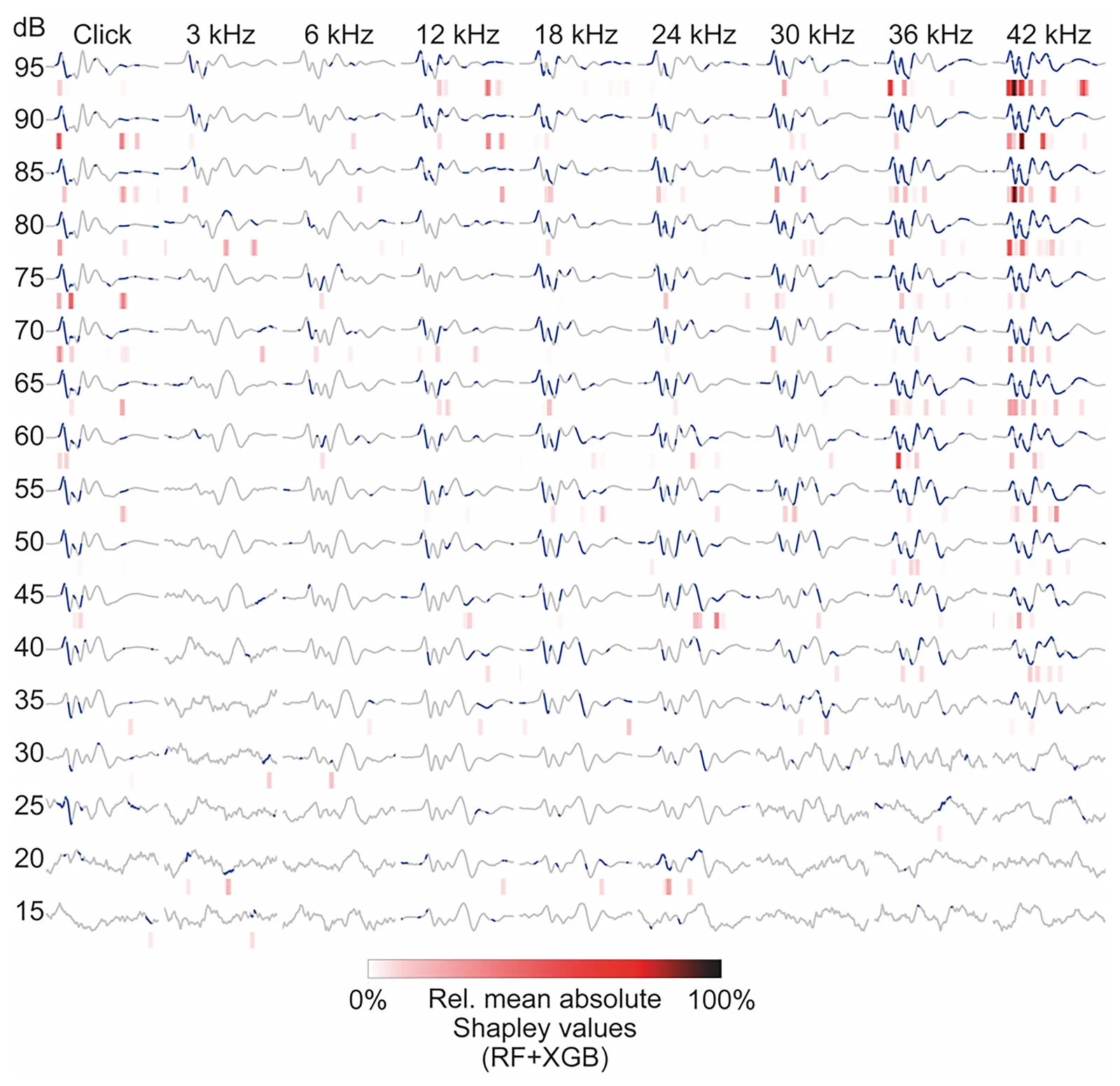
Most important features for *Cdh23*^*ahl*^ prediction highlighted by the models. Matrix of average ABR waveforms (104 mice in the primary cohort at 1-month-old from both the 6N and 6N-Repaired strains). Note that the y-axis scale is adjusted independently to the minimum and maximum for each trace. The part of the traces in blue indicates parts of the ABR selected by the ANOVA F-test feature selection step preceding the classifiers. Each trace is superimposed to a colour-coded raster plot representing the normalised mean absolute Shapley values. Higher Shapley values indicate the most influential features for model prediction. The displayed raster plots were calculated as averages of the normalised Shapley values for the random forest (RF) and XGBoost (XGB) models (see S3 Fig for the Shapley values of the two individual models). Shapley values were calculated on the test set, using the training set as background distribution. This analysis indicated that responses to higher frequencies (36 and 42 kHz) at high sound levels are the most important features for these two models, followed by features associated to click stimuli.

We then sought to train ML models through supervised learning to classify ABR recordings taken at the earliest timepoint (1 month) based on mouse strain (6N or 6N-Repaired), thereby predicting the presence of the ARHL-linked *Cdh23*^*ahl*^ allele. To demonstrate the generalisability of this approach, we tested six different ML models: four commonly used classifiers (random forest, XGBoost, support vector machine (SVM) and multi-layer perceptron (MLP)) and two time-series-specific classifiers (HIVE-COTE V2.0 and ROCKET) (see Methods for a description of each model and hyperparameter tuning procedure). All classifiers were preceded by an ANOVA F-test feature selection step, which retained 10% of the features (i.e., ABR timepoints, Fig 3a, Fig 4, see Methods). We randomly split the dataset into a train/validation set and a test set (78 and 26 mice respectively, Fig 3a). In all analyses, the “6N” strain (i.e., “mice with early-onset ARHL”) was treated as the positive class, as it represents our primary outcome of interest in evaluating model performance.

Initially, we trained the models using ABRs for the full set of sound stimuli (click and pure tones from 3 to 42 kHz) of 1-month-old mice from the primary cohort (“global” models). As input features, we concatenated the ABR waveforms recorded at various stimulus intensities and frequencies, forming a single univariate time series (Fig 1b). We first evaluated the model performances through repeated *k*-fold cross-validation on the 78 mice within the training set (*k*=5 folds and 5 repeats, for a total of 25 splits). Final scores were then calculated by averaging the results of individual splits (see Methods). We found that all tested models showed strong overall performances across recall, specificity, precision and receiver operating characteristic area under the curve (ROC AUC) metrics (Fig 3b-e, S1 Table). When focusing on recall (i.e., the true positive rate or sensitivity, reflecting the capability of the models to identify “pathological” cases), there was no significant differences among most pairs of models, except between those with the highest score (SVM) and the two tree-based models (S2 Table). Moreover, no significant differences were found in the specificity (i.e., true negative rate) and precision scores of the six models (P=0.0724 and P=0.0844 respectively, Friedman test, S2 Table). All models achieved relatively high average ROC AUC scores (Fig 3e), demonstrating strong overall discrimination ability between the classes. These results suggest that all models were generally effective in distinguishing between classes, with some models achieving higher discrimination performance on average.

The models were then trained on the whole training set (78 mice) and evaluated on the test set (26 mice, the same train/test split was kept for all models). We found that the models based on the HIVE COTE V2.0 and ROCKET classifiers showed the best performance and were able to correctly determine the strain of all the 26 mice from their ABR waveforms (100% accuracy: Fig 3f). Tree-based models (random forest and XGBoost) were slightly less accurate compared to the other four models and misclassified 4 out of 26 test mice (~15%, Fig 3f). In comparison, manual blind dataset labelling by three experimenters demonstrated varied accuracy, with each annotator mislabelling between 5 and 8 mice out of the 26 in the test set, corresponding to an error rate between ~19% and ~31%, with moderate agreement between annotators (Fleiss’ Kappa: 0.58).

When evaluated on the entire dataset (104 mice), manual classification resulted in a recall of 66.7% ± 13.6%, specificity of 82.7% ± 18.9%, and precision of 81.9% ± 14.1% (*n* = 3 annotators). The lower performance of manual classification can be attributed to its reliance on differences in high-frequency ABRs between the two genotypes. However, at the early age considered, the substantial overlap between the two genotypes reduces the reliability of human-extracted features for accurate genotype differentiation. Notably, at 1 month of age, 25 out of 50 6N mice (50%) had thresholds at 42kHz that were superimposed to Repaired mice (35 to 60 dB SPL, Fig 2a). Moreover, there was substantial overlap in the distribution of wave amplitudes and latencies (see Fig. 6 below). Therefore, human-extracted features may not sufficiently capture the subtle differences between genotypes at this early age since, unlike classification algorithms, manual classification is limited to lower-dimensional representations of the data.

To gain an insight into the ML algorithm decision process, we determined the contribution of individual features to the classification task by calculating the mean absolute Shapley values (Lundberg & Lee, 2017). We selected the random forest and XGBoost classifier for this task, as calculating Shapley values was computationally prohibitive for the other four models. We found that features corresponding to higher frequency stimuli (36 and 42 kHz) were the most influential for the classification task (Fig 4, S3 Fig). Additionally, features corresponding to click responses had Shapley value elevated across different sound levels, suggesting that subtler differences between the two genotypes may exist in the ABR waveforms associated with these stimuli (Fig 4).

Overall, these results highlight the potential of ML models to outperform human experts in identifying differences in ABRs due to ARHL, offering an accurate tool for its early detection.

### 3.3 Click ABRs are sufficient to predict the presence of the *Cdh23^ahl^* allele

**Fig 5:**
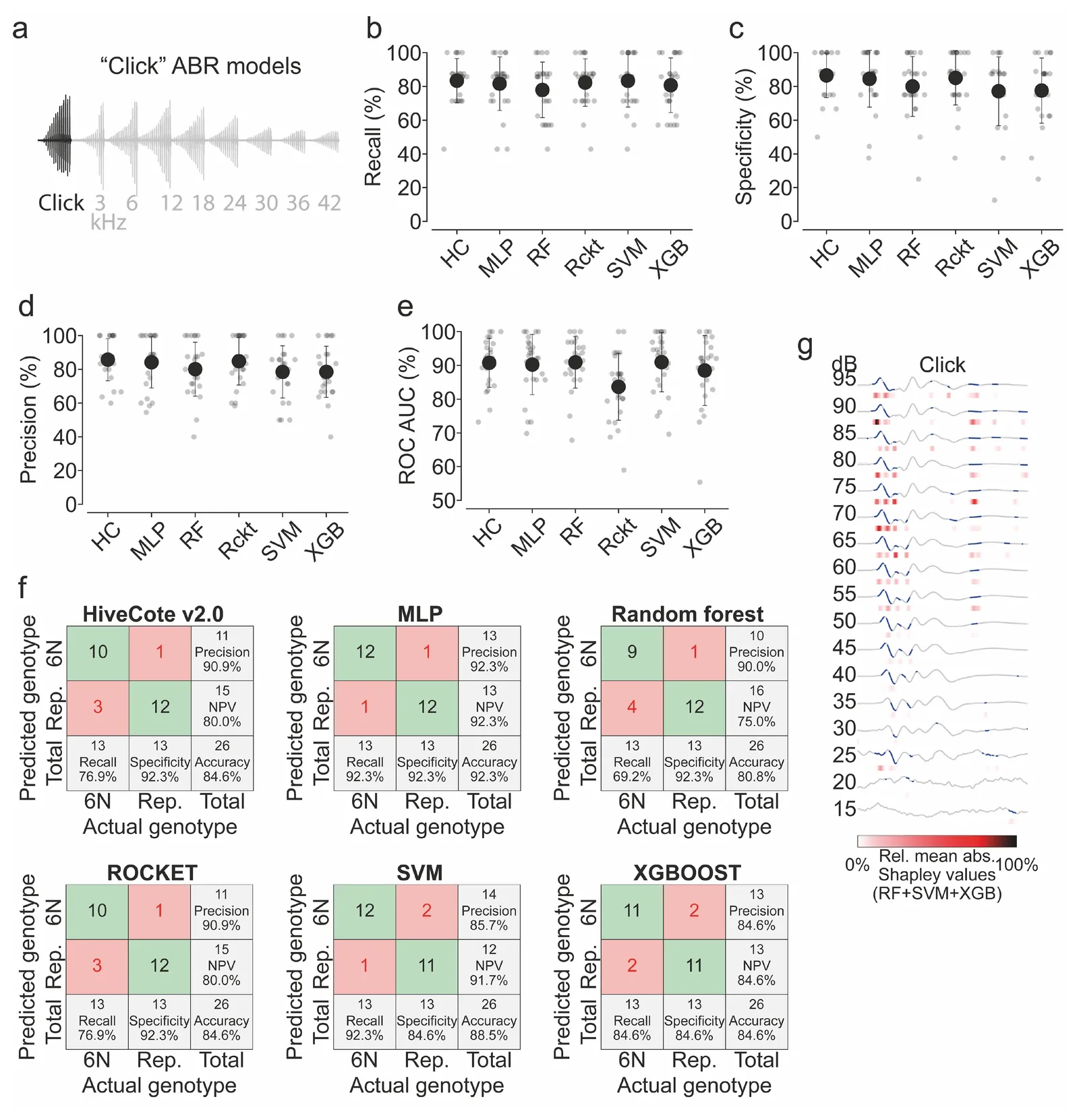
Click responses alone are sufficient for predicting the presence of *Cdh23*^*ahl*^ from ABRs (**a**) Input features for “Click” models. The greyed-out trace (i.e., tone ABRs) indicates features not used for training/testing the models (compared to “Global” models, Fig 1b, Fig 3a). (**b-e**) Average performances of the six models trained on the click ABR alone as estimated in the cross-validation step. Solid dots represent the mean ± SD. Smaller dots indicate the score of individual folds in the cross-validation step (25 scores per model, see also S3 Table and S4 Table for statistical comparisons). (**f**), Confusion matrices highlighting the performances of the models trained on the click ABR on the test set. HC: HiveCoteV2.0; MLP: multilayer perceptron. RF: random forest; Rckt: ROCKET; SVM: support vector classifier; XGB: XGBoost. Rep: 6N-Repaired. NPV: negative predictive value. (**g**) Average click ABR waveform (104 mice from 1-month-old of both 6N and 6N-Repaired strains) superimposed to a colour-coded raster plot representing the normalised mean absolute Shapley values. Shapley values from three different models (random forest, SVM and XGBoost) were normalised and averaged (see S3 Fig for the Shapley values of the individual models). The part of the traces in blue indicates parts of the ABR selected by the ANOVA F-test feature selection step preceding the classifiers. This analysis highlighted parts of wave 1 and wave 2 at sound intensities above 50 dB SPL as the features with the highest importance for model predictions.

To assess the robustness of the models under more challenging conditions, we aimed to restrict the number of input features, simulating a scenario often encountered in clinical settings where higher-frequency tone sensitivity (above 8 kHz, called the extended high frequencies, or EHF) is typically not performed (Hunter *et al*. 2020). Specifically, we asked whether the click ABR alone, which does not display any significant threshold shift until 6 months of age (Fig 2d), contained enough information to differentiate between the two mouse strains at 1 month using ML. To test this, we retrained the six models described above using only the click responses from 1-month-old mice as input features (“Click” models, Fig 5a). We found that all the models tested retained good performances across several metrics (Fig 5b-e, S3 Table, S4 Table). No significant difference was found in the recall of “Global” and “Click” models (P=0.2150, mixed-effects linear model), albeit the former were associated with higher sensitivity and precision (P < 0.0001 for both metrics) and ROC-AUC score (P=0.0330, mixed-effects linear models). When evaluated on the test set, models misclassified between 2 to 5 out of 26 mice (error rate between ~8%, MLP and ~19%, random forest) from their click ABR waveforms (Fig 5f). These results were consistent with those obtained for the “Global” models that included all frequencies tested (Fig 3). In contrast, manual annotation from the three experimenters was much less accurate when the information about high-frequency tones was removed, with each annotator mislabelling between 10 and 12 mice out of the 26 from the test set (error rates between ~38% and ~46%) with very poor agreement between annotators (Fleiss’ Kappa: -0.09).

As done previously for the “Global” models (Fig 4), we sought to interpret the “Click” models by calculating the mean absolute Shapley values for the three algorithms for which the computation was feasible on our hardware (random forest, SVM and XGBoost). We found that the mean absolute Shapley values corresponding to wave 1 and wave 2 of the click ABR waveforms were consistently elevated across the higher sound intensities (Fig 5g, S3 Fig), indicating the importance of these features for the identification of early-onset ARHL mice. Wave 1, which reflects the activity of the auditory afferent fibres, was consistently selected by the ANOVA F-test feature selection step across most intensities. This suggests that, even in the absence of an auditory threshold difference, significant variations in the output of the cochlea caused by the *Cdh23*^*ahl*^ allele may be present at an early age.

Overall, these findings indicate that ABR wave 1 features could enable ML models to distinguish between the two mouse strains at an early stage, before threshold differences emerge.

### 3.4 Differences in ABR wave 1 in 1-month-old 6N and 6N-Repaired mice

**Fig 6:**
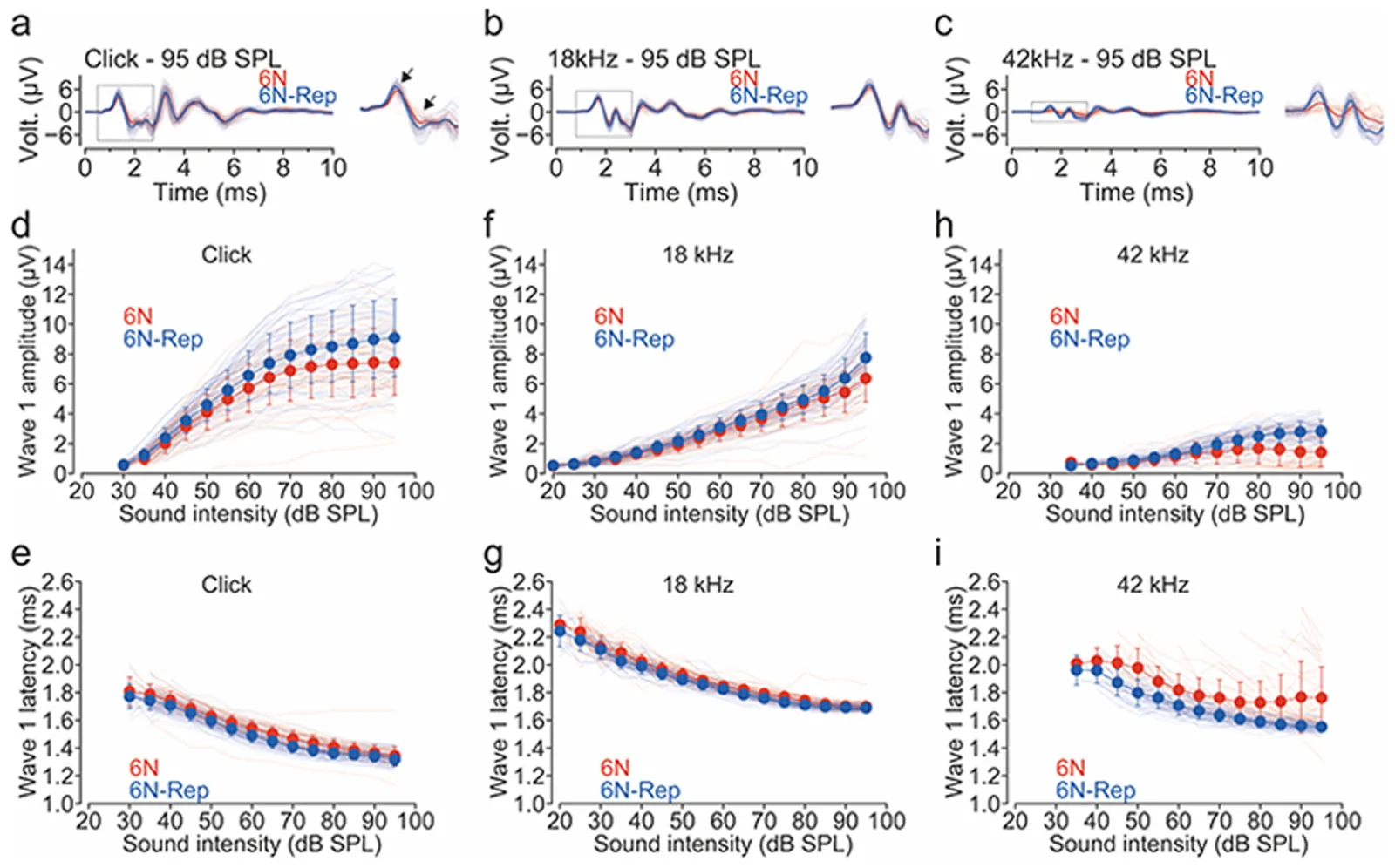
Differences in ABR Wave 1 in 1-month-old 6N and 6N-Repaired mice. (**a-c**) Comparisons of average ABR waveforms from 50 6N and 54 6N-Repaired mice for click stimuli (**a**), 18 kHz (**b**) and 42 kHz (**c**) tones at 95 dB SPL. The traces on the right provide a magnified view of the dashed in the traces on the left, highlighting the subtle differences in wave 1 between the average waveforms of the two genotypes (arrows in panel **a**). (**d-i**), Average wave 1 amplitude (**d**,**f**,**h**) and latency (**e**,**g**,**i**) as a function of sound level for 6N and 6N-Repaired mice for click, 18 kHz and 42 kHz sound stimuli. Significant differences between the two mouse strains: P < 0.0001 (for the three stimuli for both amplitude and latency, two-way ANOVA, panels **d-i**). Solid lines represent the mean ± SD, while lighter traces show individual mice.

Next, we tested whether the importance of features in wave 1, which were used by some models to identify 6N and 6N-Repaired mice, were underpinned by differences in the average wave 1 amplitude and latency between the two strains. Using ABR click responses from 1-month-old mice, we found that both wave 1 amplitude and latency differed significantly between the two mouse strains, despite substantial overlap in their distributions (Fig 6a,d,e). The difference in average amplitude was maximal at 95 dB SPL (1.6 µV, 18.4%), while the difference in average latency was maximal at 70 dB SPL (56 µs, 3.9%). Significant differences were also found in latency and amplitude in the individual tone responses (e.g., 18 kHz: Fig 6b,f,g and 42 kHz Fig 6c,h,i, see also S4 Fig). These results indicate that, in a mouse model of early onset progressive hearing loss, ABR waveforms may undergo subtle changes well before a more obvious threshold shift appears. These changes can be detected by ML models, potentially identifying early hallmarks of the dysfunction.

To further investigate the role of these features in classification, we compared the performance of a support vector machine (SVM) when trained on only wave 1 latencies and amplitudes or auditory thresholds, compared to when the full ABR waveform was used as input (S5 Fig). This analysis was performed on both a “Global” dataset (including wave 1 parameters and thresholds for all click and pure-tone stimuli) and a “Click” dataset (i.e., using only waveforms, thresholds, wave 1 amplitudes and latencies from “Click” ABRs). We found that using the full ABR trace led to significantly higher recall and ROC-AUC scores, reflecting both improved sensitivity in detecting mice carrying the ARHL-linked allele and better discrimination between the two classes across different probability thresholds. Moreover, the full-trace model consistently outperformed wave 1 and threshold-based models on the test dataset, with all models trained using the same train/test split and cross-validation folds (S5 Fig). Taken together, these findings indicate that allowing the model to autonomously determine the most relevant features may offer advantages over hypothesis-driven feature selection, leading to improved classification performance.

### 3.5 ML models performances on heterogeneous datasets

**Fig 7:**
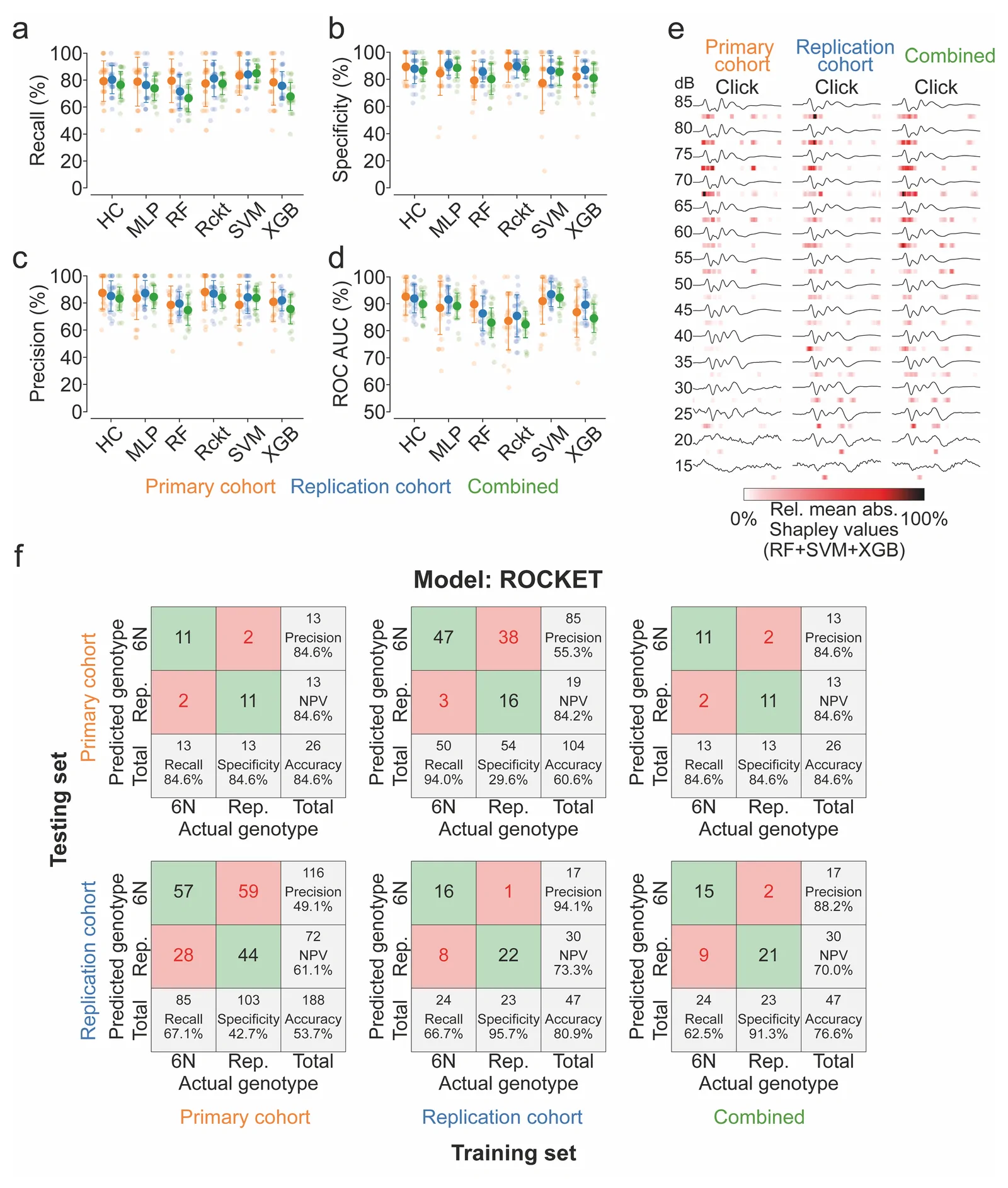
ML model performances decrease when predicting an external dataset. (**a-d**) Average metrics of the six models trained on click ABR as estimated in the cross-validation step. Models were trained and validated on each of two independently acquired datasets (primary and replication cohort) or on a combined dataset from one-month-old mice. To align with the acquisition protocols of the two datasets, only sound intensities from 15 dB SPL to 85 dB SPL were used. Solid dots represent the mean ± SD while the smaller dots indicate the scores from individual folds in the cross-validation step (25 scores per model per dataset, see also S5 Table). (**e**) Average click ABR waveforms from three datasets (primary cohort, replication cohort, combined) superimposed to a colour-coded raster plot representing the normalised mean absolute Shapley values. The values of three different models (random forest, SVM and XGBoost) were normalised and averaged. Models were trained and tested on either the primary cohort dataset (left), replication cohort dataset (center) or both datasets combined (right). This analysis consistently identified parts of wave 1/ wave 2 as the features with the highest importance for model predictions across datasets. Primary cohort: 50 6N and 54 6N-Repaired (Rep.) mice; replication cohort: 85 6N and 103 6N-Repaired mice. (**f**) Array of confusion matrices displaying performances of ROCKET models with different combination of the three datasets used for training and/or testing. For tasks which involved evaluating the models on data from different laboratories (i.e. training on replication cohort/testing on primary cohort or vice versa), the whole primary and replication cohort datasets were used either for training or testing. Note that results for training/testing on primary cohort data (top left confusion matrix) are slightly different from the same model in Fig 5f due to the difference in input features between the two (15-95 dB SPL click ABRs vs 15-85 dB SPL click ABRs). HC: HiveCoteV2.0; MLP: multilayer perceptron. RF: random forest; Rckt: ROCKET; SVM: support vector classifier; XGB: XGBoost. Rep: 6N-Repaired. NPV: negative predictive value.

To test the ability of the models to generalise to a similar set of ABR data obtained from a different experimental setting, we replicated the previous analysis incorporating into the training/testing data an independently acquired ABR dataset (**replication cohort**, Fig 7). This dataset contained 188 click ABRs of 1-month-old mice (85 6N and 103 6N-Repaired) and, differently from the primary cohort dataset (Figs 1–6), was obtained from mice of both sexes.

We first tested whether the general approach described above was also applicable to the replication cohort by retraining the ML models using either the primary or the replication cohort datasets, or the two sets combined for training/validation (Fig 7a-d, S5 Table). As the replication cohort dataset contained ABRs for stimuli up to 85 dB SPL, we retrained the models from Fig 5 using only this subset of sound intensities from the primary cohort dataset to allow for comparison. Across the main four metrics (recall, sensitivity, precision, and ROC AUC), no statistically significant differences were found between datasets (primary, replication, or combined cohorts, P > 0.1 for all comparisons, mixed-effect linear model, Fig 7a-d). Moreover, feature importance analysis using Shapley values highlighted similar features in the primary and replication cohorts (both alone and combined), roughly corresponding to wave 1 and wave 2 (Fig 7e) as previously shown (Fig 5g). Overall, this result highlights the generalisability of our ML-based approach in capturing relevant patterns in ABR data for the identification of early hallmarks of *Cdh23*^*ahl*^-related ARHL.

Next, we measured the performance of one of the models (ROCKET) in making predictions on a dataset different from the one it was trained on (Fig 7f). We found that the accuracy greatly decreased when making predictions on a different set. A model trained on the primary cohort dataset correctly classified only 101 out of 188 mice (54%) from the replication cohort (recall: 67%, specificity: 43%), while a model trained on 75% of the replication cohort dataset correctly predicted the genotype of 63 out of 104 mice (61%) from the primary cohort (recall: 94%, specificity: 30%, Fig 7f). In contrast, accuracy on held out test data of either cohort remained higher when the model was trained on a combined dataset (Fig 7f). These findings suggest that incorporating data from multiple sources during training is essential to maintain a high prediction accuracy.

### 3.6 ML based prediction of the progression of hearing function in mice

**Fig 8:**
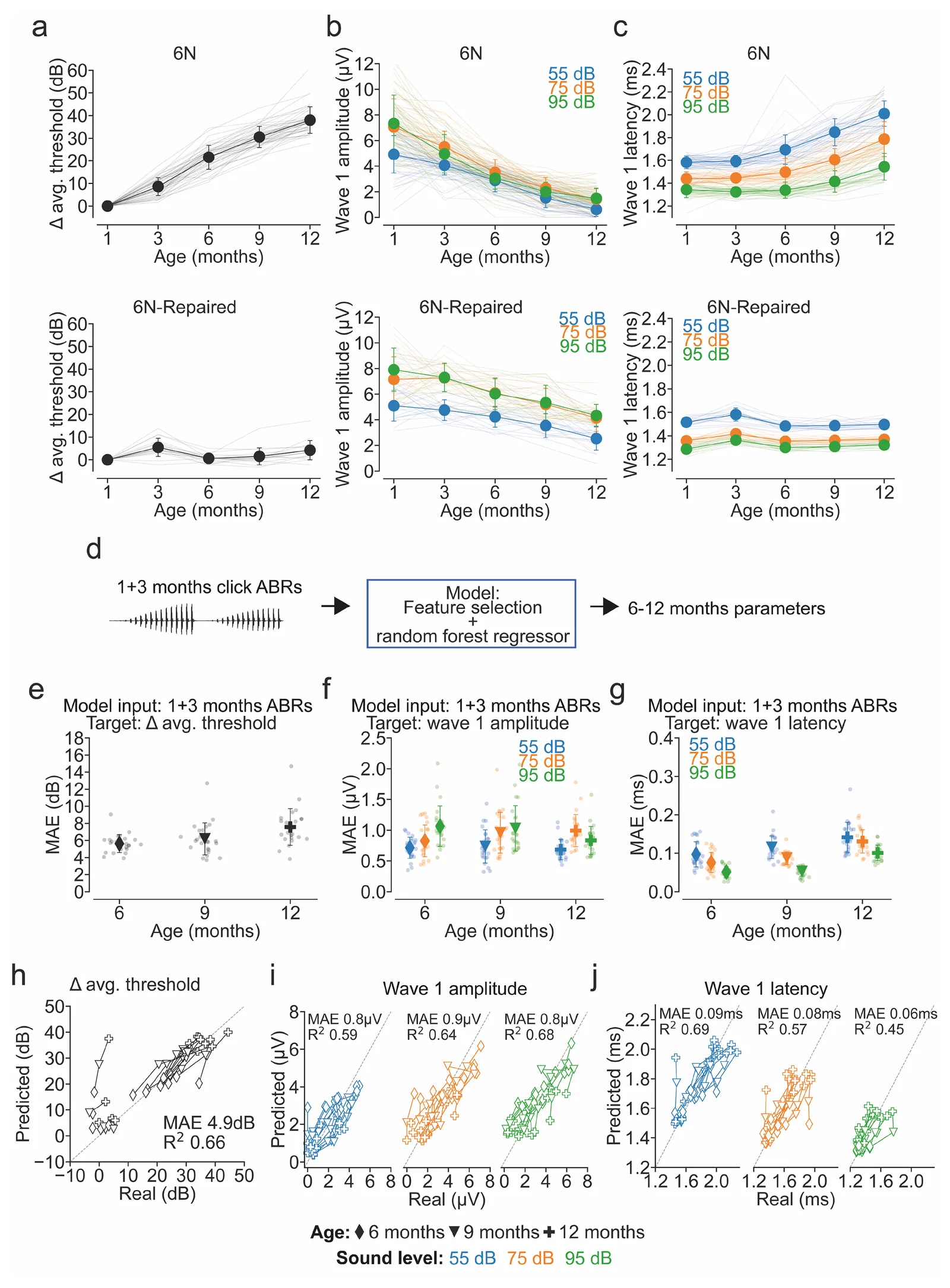
ML approach to predict the progression of hearing function in mice. (**a-c**) Change in ABR properties over time for 6N (top panels) and 6N-Repaired (bottom panels) mice (45 6N and 18 6N-Repaired mice). Panel (**a**) displays the change in average threshold, calculated as the difference between the average thresholds for click and eight tones relative to the value at 1 month of age. Wave 1 amplitude and latency for click stimuli at three different sound levels (55 dB, blue; 75 dB, orange and 95 dB, green) are shown in panels (**b**) and (**c**), respectively. (**d**) Scheme of the regression model. The models included an ANOVA feature selection step, selecting 10% of timepoints as features (see Methods), followed by a random forest regression model. The input of the models consisted of concatenated click ABRs at 1 and 3 months of age, while the targets were the values of the three ABR parameters described in panels (**a-c**) at 6, 9 and 12 months of age. Training/cross validation set: 47 mice; test (hold-out) set: 16 mice. The same training/validation/testing split was kept for all the models. (**e-g**) Mean absolute error (MAE) of the regression models for changes in average thresholds (**e**), wave 1 amplitude (**f**) and latency (**g**) of click stimuli, as estimated in the cross-validation step. The target age is indicated on the x axis. Filled dots represent the mean ± SD. Smaller dots show the score of individual folds in the cross-validation step (5 folds, 5 repeats, 25 scores per age and sound level). (**h-j**) scatter plots displaying predicted versus real values for the change in average threshold (**h**) and wave 1 amplitudes (**i**) and latencies (**j**) at the indicated sound levels for the test set (16 mice). Each triplet of connected symbols represents an individual mouse in the test set. The coefficient of determination (R^2^) and the mean absolute error (MAE) of each model are indicated at the top. The dashed grey lines represent ideal predictions.

We next examined whether ABR waveforms from young adult mice contained information to predict the future progression of their hearing function. To do so, we used data from the 63 mice (45 6N and 18 6N-Repaired mice) from the primary cohort for which ABR measurements were collected at 1, 3, 6, 9 and 12 months of age. ABRs of 6N-Repaired mice were included in the training set to expose the models to data from “good hearing” mice, thus providing a wider range of targets. We sought to train models to predict three parameters: the shift in average thresholds across all stimuli (Fig 8a), and wave 1 amplitude (Fig 8b) and latency (Fig 8c) for click stimuli at three different sound pressure levels (55, 75 and 95 dB SPL). These parameters showed significant age-dependent changes in 6N mice (shift in average threshold: P < 0.0001, one-way ANOVA; wave 1 amplitude and latency: P < 0.0001 for both, two-way ANOVA). For example, the average threshold increased by 38.0 ± 5.9 dB SPL from 1 to 12 months in 6N mice, compared to an increase of only 4.2 ± 4.3 dB SPL in 6N-Repaired mice (Fig 8a). Moreover, substantial variability in the progression of these parameters was observed across 6N mice (Fig 8 a-c, see also S6 Fig).

We trained regression models through supervised learning using click ABR waveforms from 1- and 3-month-old mice as input features, while the values of the parameters mentioned above at 6, 9 and 12 months were used as targets (Fig 8d). We randomly divided the dataset into a train/validation set and a test set (47 and 16 mice respectively; the same training and test mice were kept across all models). As above, we first evaluated model performances through repeated *k*-fold cross-validation by averaging the mean absolute error (MAE) across splits (*k*=5 folds and 5 repeats, totalling 25 splits, Fig 8e-g, see Methods). We found that the wave 1 latency model (Fig 8g) was the most accurate in predicting the target values (the average MAE was between 3.9% and 7.9% of the mean wave 1 latency values at the corresponding age and sound levels, Fig 8c). When evaluated on the test set, the models exhibited similar MAE to those calculated in cross-validation, with a coefficient of determination (R^2^) ranging from 0.45 to 0.69 (Fig 8h-j).

## 4 Discussion

The application of machine learning (ML) to large biomedical datasets is expected to drive profound changes in clinical diagnosis, delivery of precision medicine and health monitoring (Goecks et al., 2020). In this study, we applied ML to identify early signs of hearing loss and to predict its progression in mice using ABR waveforms as input data. We tested six different ML algorithms on the task of classifying which mice carried *Cdh23*^*ahl*^ (6N) compared to co-isogenic *Cdh23* repaired mice (6N-Repaired), at a time when hearing thresholds are similar between the two mouse strains (Johnson *et al*. 1997; Noben-Trauth *et al*. 2003; Mianné *et al*. 2016; Jeng *et al*. 2020a; 2020b). We tested both widely used classifiers (e.g., random forest and SVM) and state-of-the-art algorithms specialised for time series classification (HIVE-COTE V2.0 and ROCKET). The models we implemented retained very good performances even when a restricted set of data (click ABRs) was used instead of the full range of information (pure tone ABRs). This indicates that ML algorithms were able to identify key features associated with hearing loss even in the absence of differences in ABR thresholds, which can more easily be identified by trained experimentalists. None of the six models tested demonstrated a clear advantage in the classification task. Time-series specific classifiers like HIVE COTE V2.0 and ROCKET were the most consistent across different tasks, albeit with longer computational analysis time compared to the other models. In contrast, tree-based models (Random Forest and XGBoost) were less effective, despite offering easier interpretability of their decision processes. Overall, even simpler models, like the one based on the SVM classifier, performed reasonably well in the classification task, suggesting that more complex models might not provide a substantial improvement over simpler ones. However, the consistency of results across different models strengthens the validity of our approach and supports its robustness.

Interpretation of the models’ decision process for the identification of mice carrying the *Cdh23*^*ahl*^ allele revealed that the most important features were associated with wave 1, in agreement with the mutation being located in a gene expressed in the cochlear hair cells. Indeed, wave 1 was consistently selected by the ANOVA F-test feature selection step preceding the classifiers, and its amplitude and latency were significantly different between the two genotypes already at one month of age. We validated our ML approach by applying it to a second, more heterogeneous, ABR dataset acquired by another laboratory. We also demonstrated that ML models are well suited to predict the future trajectory of hearing capabilities in mice from early timepoints.

### 4.1 Early hallmarks of progressive hearing loss in mice with *Cdh23^ahl^*

In mammals, acoustic information travelling within the cochlear partition is transduced into a receptor potential in the sensory hair cells by the mechanical displacement of the stereociliary bundles projecting from their apical surface (Fettiplace, 2017). Within each hair bundle, individual stereocilia are interconnected by several extracellular linkages (Tilney *et al*. 1992; Goodyear *et al*. 2005). One of these linkages, the tip link, is formed by cadherin 23 and protocadherin 15 (Siemens *et al*. 2004; Ahmed *et al*. 2006; Kazmierczak *et al*. 2007). Tip links transmit force generated during sound-induced displacement of the hair bundles to open mechanoelectrical transducer (MET) channels located at the tips of the shorter rows of stereocilia (Beurg *et al*. 2009). MET channel opening leads to the depolarization of hair cells, and fusion of glutamate-filled synaptic vesicles at ribbon synapse active zones, which allow high-rate synaptic transmission onto the auditory afferent fibres (Glowatzki & Fuchs, 2002; Keen & Hudspeth, 2006; Goutman & Glowatzki, 2007).

The widely used C57BL/6 mice have a single-nucleotide polymorphism in exon7 of the gene encoding cadherin 23, which affects splicing and leads to skipping of exon 7 (*Cdh23*^*ahl*^, Johnson *et al*. 2017; Noben-Trauth *et al*. 2003). *Cdh23*^*ahl*^ has been shown to cause hearing loss starting in the high-frequency cochlear region by about 3 months of age, and then progressing towards the low-frequency, so that C57BL/6 mice become almost completely deaf by 12-18 months of age (Johnson *et al*. 1997; Jeng *et al*. 2020a; Jeng *et al*. 2020b; Jeng *et al*. 2021; Kane *et al*. 2012; Peineau *et al*. 2021). Sensitive high frequency ABR thresholds are maintained into old age in co-isogenic repaired C57BL/6 mice (6N-Repaired), in which the *Cdh23*^*ahl*^ allele was repaired with targeted CRISPR/Cas9 gene editing (Mianné *et al*. 2016). The progression of hearing loss of C57BL/6N mice used in this study is consistent with the above previous investigations when using a similar ABR threshold detection approach. However, the larger sample size of our dataset used to train ML algorithms allowed us to identify small differences in the ABR waveform of 6N compared to 6N-Repaired mice already at 1 month of age, which is a time when both mouse strains are considered to have normal hearing (Fig 6). These differences included a significant decrease in the amplitude and increase in the latency of wave 1, which is determined by the synchronous activity of auditory nerve fibres, and it is generally interpreted as a measure of the neural output of the cochlea. Our results are therefore consistent with the idea that a reduction in cochlear output is an early sign of the auditory decline associated with ARHL, preceding threshold elevation (Sergeyenko *et al*. 2013). Moreover, this work indicates that age-related changes to hair cell function in mice harbouring the *Cdh23*^*ahl*^ allele may occur earlier than previously observed based on changes in synapse count and morphology (Jeng *et al*. 2020b; Peineau *et al*. 2021; Stamataki *et al*. 2006). Further investigations will be required to determine the physiological correlates of this reduction.

### 4.2 Wave 1 as an early predictor of progressive hearing loss due to cochlear impairment

The close correspondence between wave 1 and synaptic survival (Kujawa & Liberman 2009) indicates that non-invasive measures of cochlear neural responses, such as the one provided by ABR tests, are a suitable method for early diagnosis of hearing loss even in the presence of normal thresholds, as previously suggested (hidden hearing loss, Sergeyenko *et al*. 2013). Our work demonstrates that ML algorithms can “learn” to single out early differences in ABR data without any a-priori hypothesis of the features that are indicative of hearing loss. The number of features extracted by computational algorithms far exceed those that can be identified by trained experimenters/clinicians (e.g., thresholds, absolute amplitudes and latencies, wave 5/1 ratio and interwave 1-5 latency, Verhulst *et al*. 2016). Moreover, our work demonstrates that feature selection approaches that rely on predefined parameters, such as wave 1 amplitudes and latencies or auditory thresholds, can be less sensitive in identifying early-onset ARHL cases. Conversely, a data-driven approach, in which the model autonomously determines the most relevant features, improves classification performance compared to hypothesis-driven feature selection. Our findings align with prior work showing the potential of machine learning to enhance the objectivity and accuracy of ABR waveform classification (McKearney & MacKinnon 2019). Moreover, ML applications are recently making significant progress in hearing healthcare and research (Lesica *et al*. 2021; Chen *et al*. 2021; Shew *et al*. 2019, Cha *et al*. 2019, Crowson *et al*. 2023, Chen *et al*. 2024).

One shortcoming of our approach is that our models demonstrated poor performance when tested on mice from a different cohort, highlighting a key limitation in generalizability and indicating a tendency to overfit to the specific domain used for training. The poor performances are likely linked to variations in equipment and techniques used in ABR recordings, such as electrode placement, mouse position in respect to the speakers, variations in preprocessing pipelines or subtle differences in task execution by the experimenters, leading to a distribution shift between the two datasets. However, we found that incorporating labelled data from the target cohort (Combined dataset) in the training phase was sufficient to maintain classification performance. In the absence of labelled data from the target cohort, transfer learning techniques (e.g. Azab et al. 2019; Azab et al. 2020, Giles et al. 2022) could be used to merge knowledge from data collected in different settings to boost model performance across different data sets. For example, domain adaptation techniques such as Domain-Adversarial Neural Networks (DANN, Ganin *et al*. 2016) or CORAL (Sun *et al*. 2017), could help mitigating this issue by aligning feature distributions across datasets, potentially improving model robustness. However, these methods rely on neural networks and are therefore likely to require larger datasets than those used in this study.

Furthermore, the models were trained exclusively on mice carrying the *Cdh23*^*ahl*^ allele and co-isogenic controls, limiting their generalizability to other pathologies affecting ABR waveforms, including other forms of progressive hearing loss. In order to improve the robustness and applicability of the model, future work should focus on expanding the dataset to include a broader cohort of mice, encompassing different ages and genetic backgrounds. Transfer learning techniques could also facilitate the extension of models to the diagnosis of other hearing pathologies associated with changes in ABR waveforms (e.g. Schaette & McAlpine 2011). A potential approach to further improve model robustness could involve using individual ABR trials rather than averaged waveforms to train/test models, as done in the present work. Resampling individual trials could also provide a more in-depth assessment of the performance of the models under varying conditions.

We demonstrated that ML is able not only to identify early signs of hearing loss due to the *Cdh23*^*ahl*^ allele, but also forecasting the future progression of hearing loss in mice. Interestingly, forecasting the progression of hearing loss was recently applied to a longitudinal study with patients affected with *GJB2*-related sensorineural hearing loss (Chen *et al*. 2024). However, translating this approach to humans with unknown mutations linked to hearing loss would face numerous challenges. Variability in ABR waveforms is notably higher in human data than in controlled animal models. Moreover, amplitudes and latencies can vary significantly, both within a single clinic and even more so across different clinics. Electrode montage around the patient head and head size are also known factors influencing ABR measurement in humans (King & Sininger 1992; Mitchell *et al*. 1989). These challenges could be overcome by developing a ABR testing pipeline that will allow the acquisition of high-quality, standardised, well curated ABR datasets. Moreover, wave 1 is usually small and more difficult to identify in humans than in mice (Bramhall 2021). Therefore, other non-invasive measurements of auditory nerve activity, such as the auditory nerve compound action potential recorded with an extra-tympanic electrode (Eggermont 2017) could be used to develop ML-based diagnostic tools. Finally, multimodal machine learning models that integrate both structured (e.g., age, gender (Jerger & Johnson, 1988)) and unstructured data (e.g., clinical notes) may be required to achieve reliable predictions.

An ML-based approach could also be applied to the identification of new genes involved in progressive auditory dysfunction in large-scale screening studies, which often rely on thresholds as the primary metric (Bowl et al. 2017; Ingham et al 2019). These, however, are relatively insensitive to primary neuronal degeneration without hair cell loss (Kujawa & Liberman 2009). By contrast, an ML-based approach could provide a more sensitive tool that does not depend on human-labelled parameters indicative of hearing loss, potentially enabling the discovery of new genes implicated in ARHL.

Overall, our findings demonstrate the potential of machine learning applied to ABR data for early detection of hearing loss, providing a framework for developing more sensitive, comprehensive diagnostic tools. While our study focused on a controlled mouse dataset, future work will be necessary to assess the applicability of this approach to human data, where ABR variability across clinics and individuals presents additional challenges.

## Supplementary Material

Supplementary data

## Data Availability

The data that support the findings is available upon request. The code is available on GitHub at https://github.com/fedeceri85/abr-ml-analysis-paper.
