# Supplementary material for: A machine-learning-based approach to predict early hallmarks of progressive hearing loss
Source: Hear Res. Author manuscript; Available in PMC 2026 Oct 27. (PMC7619641; doi:10.1016/j.heares.2025.109328)
Supplement: Supplementary file 1 [file EMS214138-supplement-Supplementary_data.pdf]

A machine-learning-based approach to predict early hallmarks of progressive hearing loss.

Federico Ceriani, Joshua Giles, Neil J Ingham, Jing-Yi Jeng, Morag A Lewis, Karen P Steel, Mahnaz Arvaneh, Walter Marcotti

This document includes:
- Six Supplementary Figures
- Six Supplementary Tables

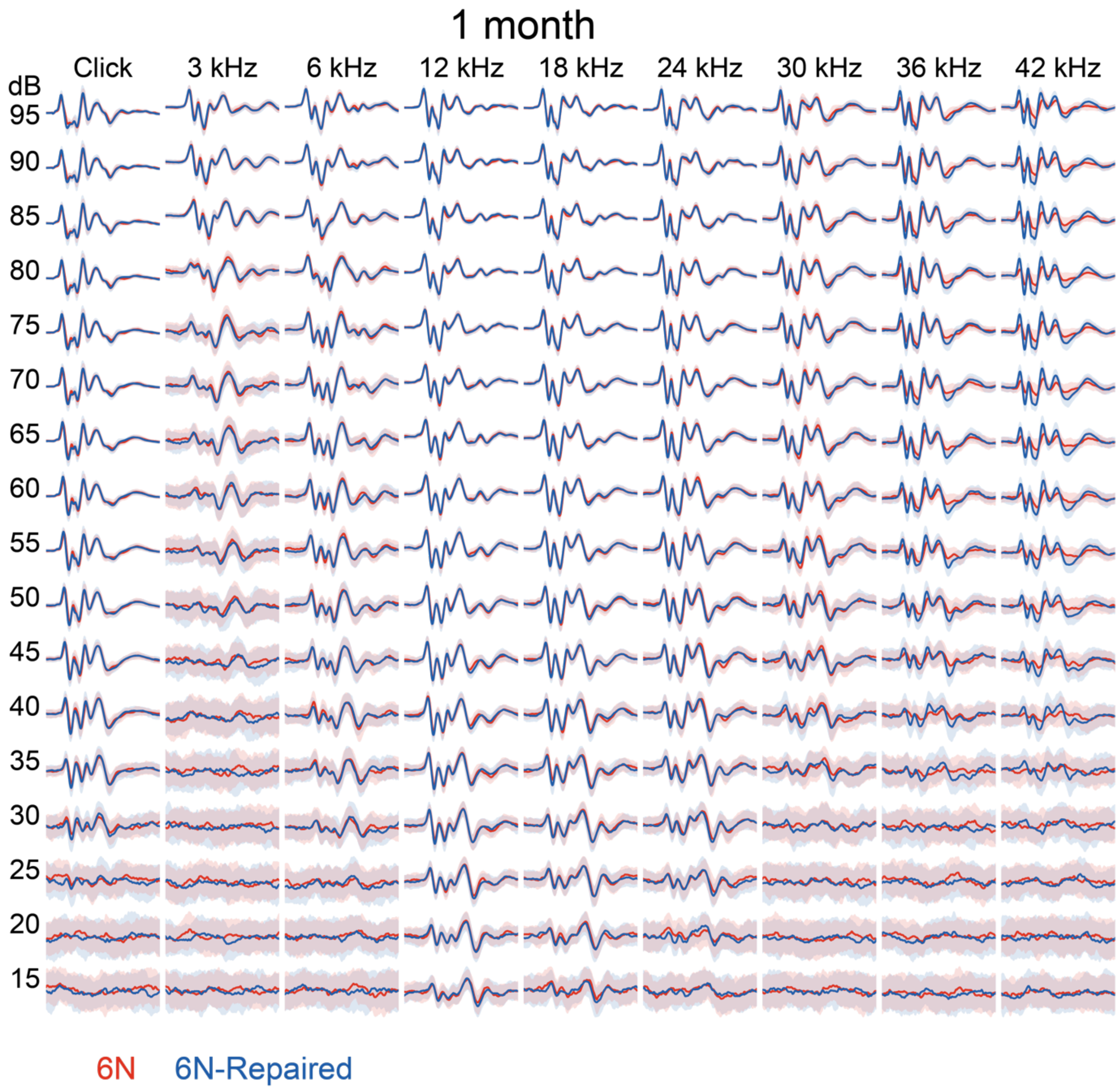

S1 Fig. Average ABR waveforms of 1-month-old mice.

Solid lines represent the average waveform while the shaded area is the SD. Note that the y-axis scale is adjusted to the minimum and maximum for each trace separately. 50 6N and 54 6N-Repaired mice.

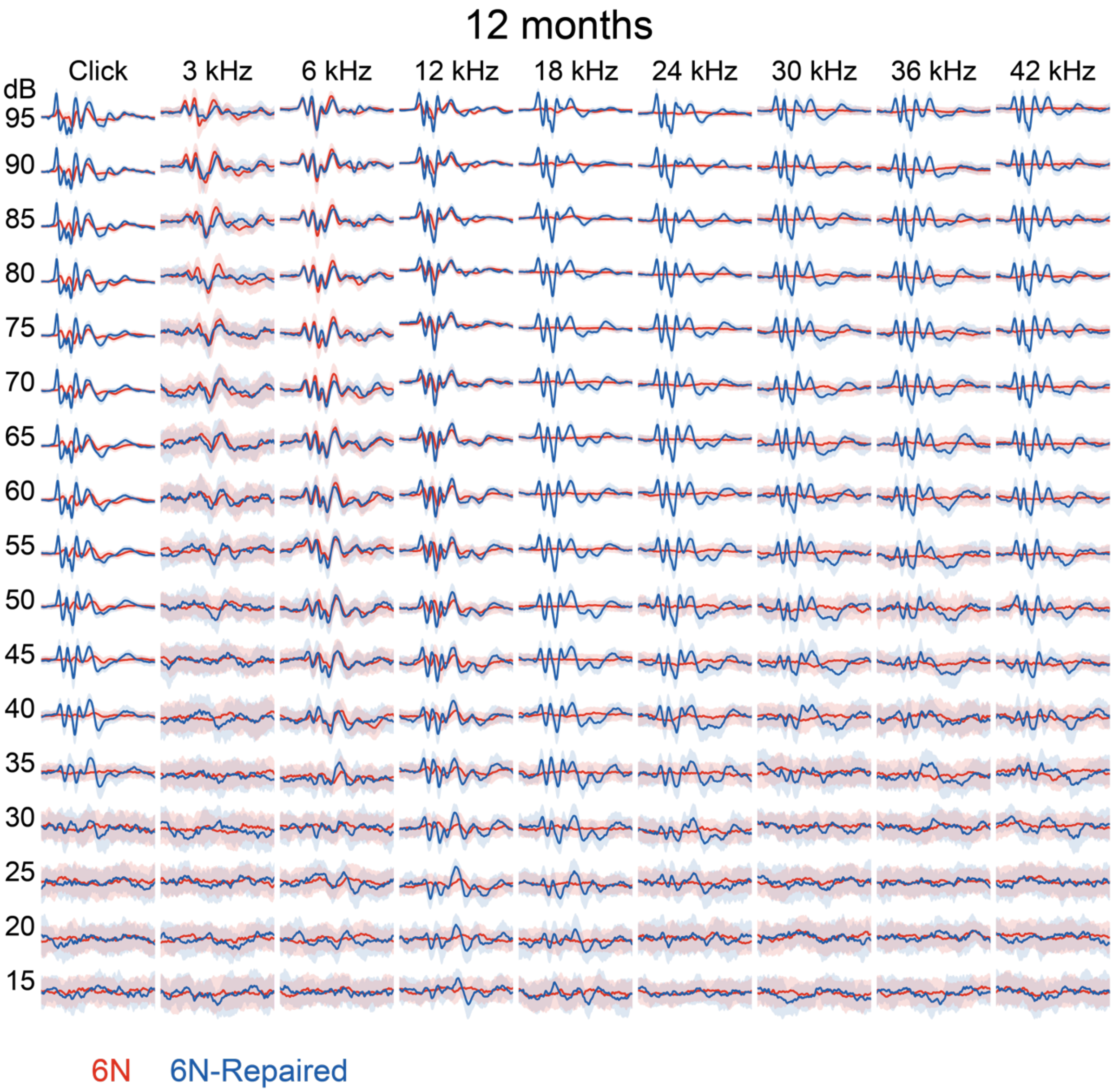

S2 Fig. Average ABR waveforms of 12-month-old mice.

Solid lines represent the average waveform while the shaded area is the SD. Note that the y-axis scale is adjusted to the minimum and maximum for each trace separately. 45 6N and 18 6N-Repaired mice.

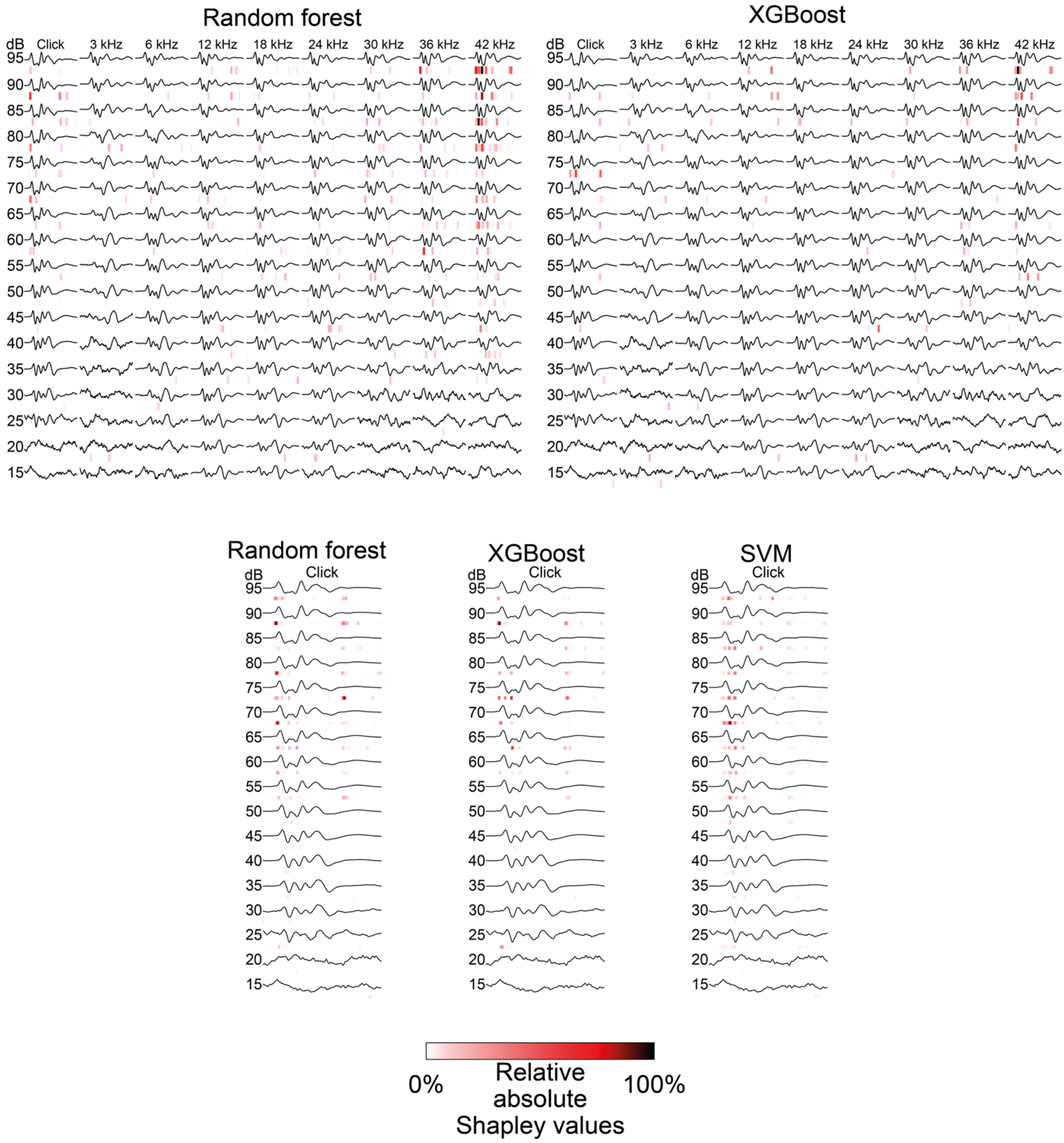

S3 Fig. Shapley values for individual models.

ABR waveforms superimposed to a colour-coded raster plot representing the normalised absolute Shapley values for “Global” models based on Random forest and XGBoost classifiers (top) and Random forest, XGBoost and SVM classifiers for “Click” models (bottom).

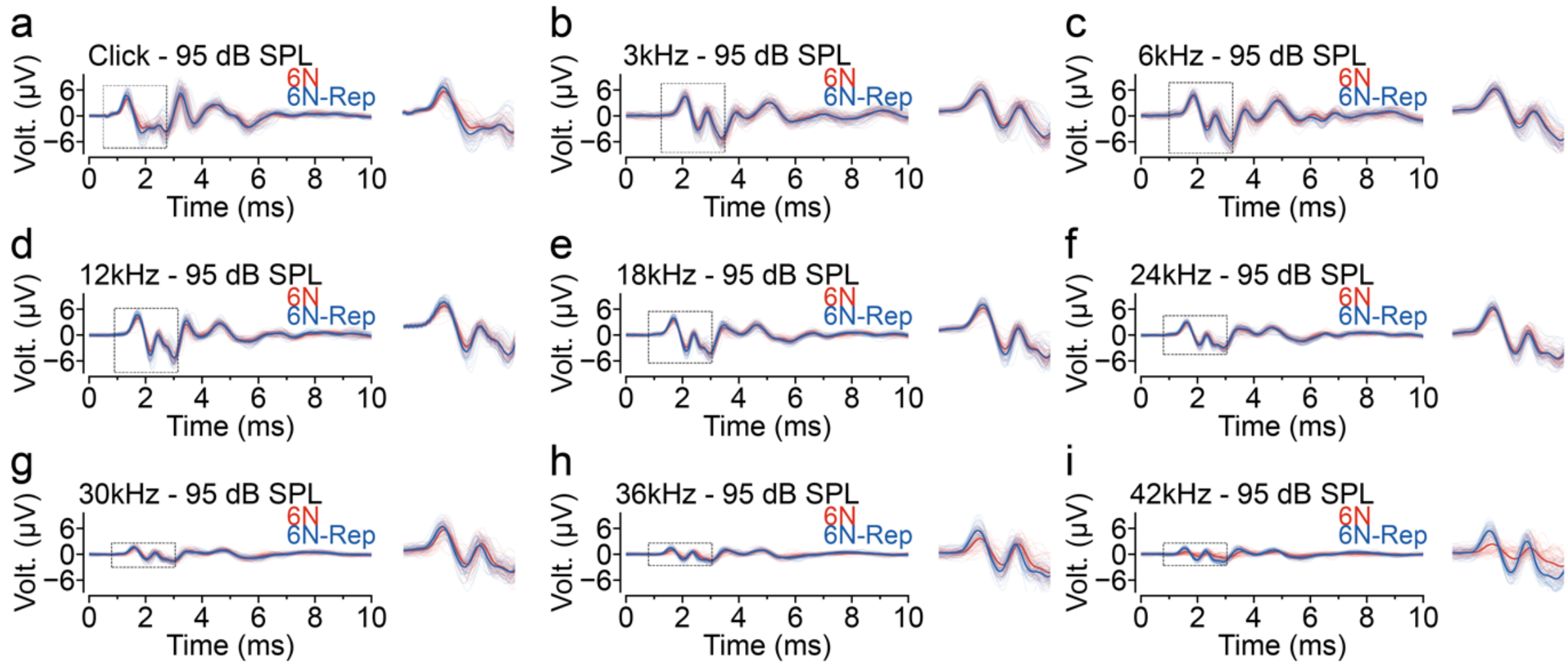

S4 Fig. Differences in ABR wave 1 in 1-month-old mice.

(a-i) Comparisons of average ABR waveforms (50 6N and 54 6N-Repaired mice) for click stimuli (a) and pure tones (b-i) at 95 dB SPL. Traces on the right represent a magnification of the dashed box drawn on the traces on the left.

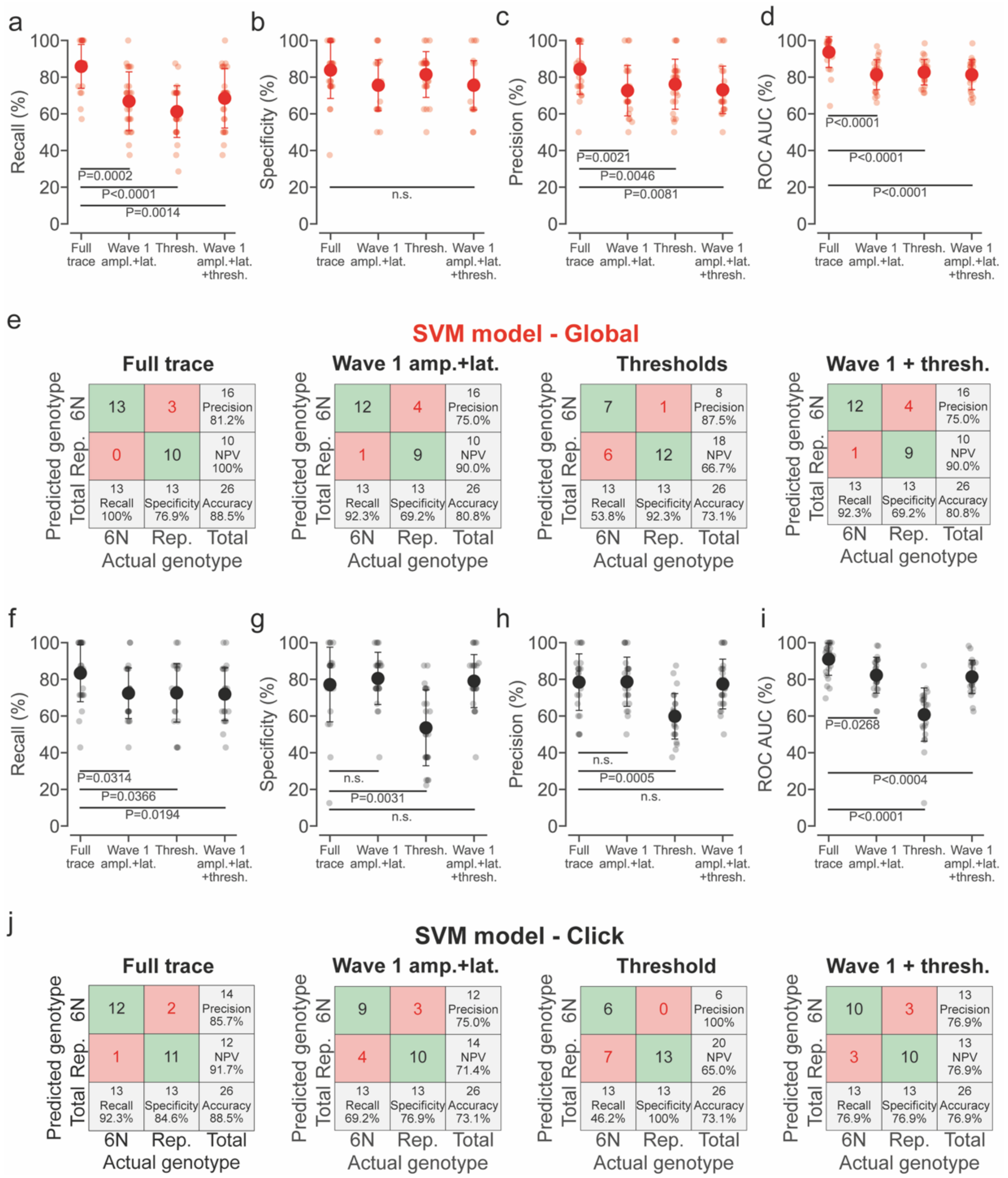

S5 Fig. Comparison of classifier performance for different input presentation.

(**a-d**) Average metrics of SVM classifiers trained on the whole ABR ("Full trace", results are from **Fig 3**) or on wave 1 amplitude and latency measurements or auditory thresholds, or a combination of the three. Metrics were estimated in a cross-validation step. Smaller dots indicate the scores from individual folds in the cross-validation step (25 scores per model). Statistical comparisons were made using Friedman test followed by Nemenyi post-hoc test. **(e)** Confusion matrices highlighting the performances of the models on the final test set. (**f-i**) Average metrics of SVM classifiers trained on Click ABR as described in panels **a-d**. "Full trace" results are from **Fig 5**. **(j)** Confusion matrices highlighting the performances of the models on the final test set. Solid dots in panels **a-d** and **f-i** represent the mean ± SD.

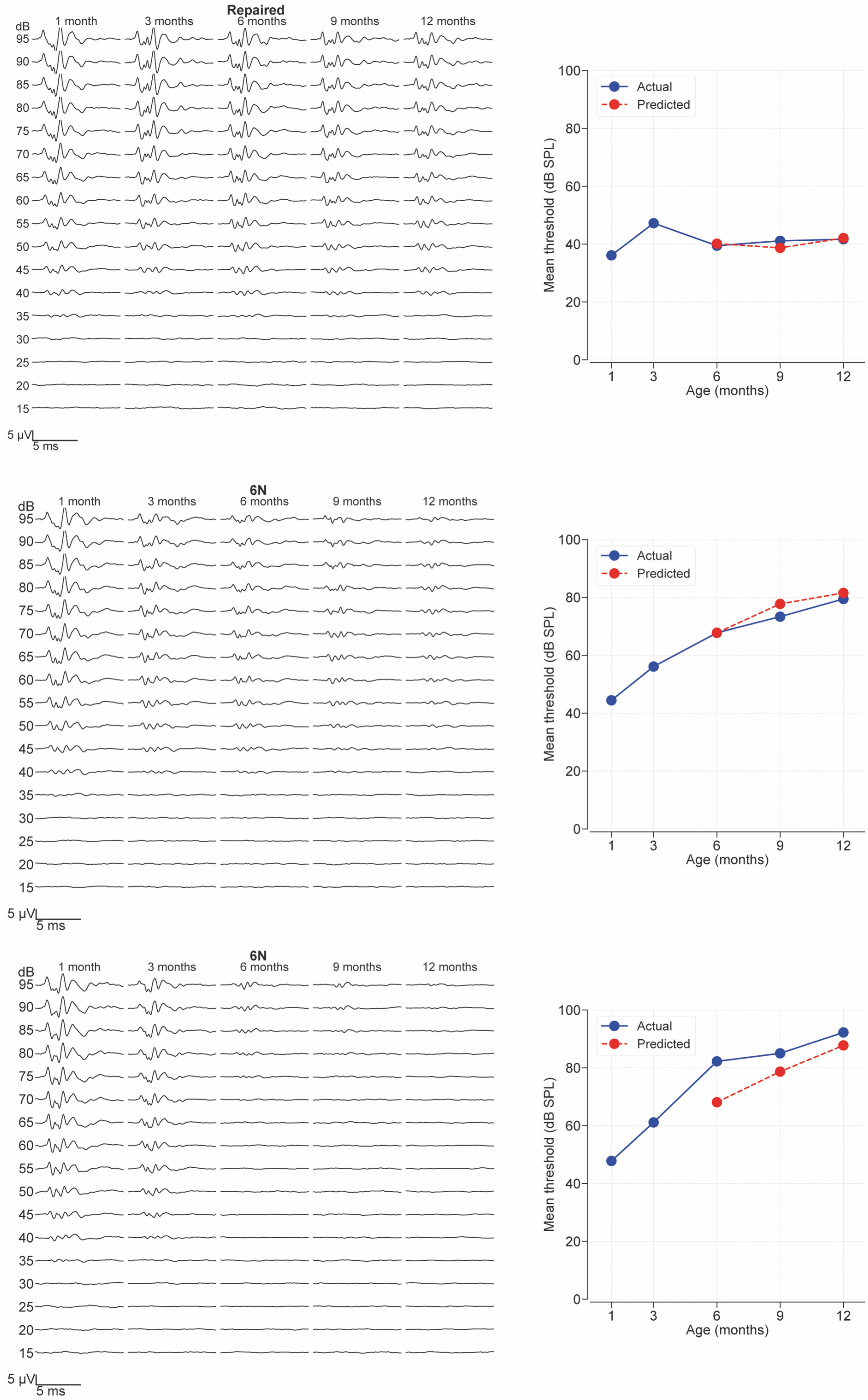

S6 Fig. Age-related changes in ABR waveforms.

Left column: sample Click ABR waveforms from 3 mice (two 6N and one Repaired) displaying the progressive age-related changes over 12 months. Right column: comparison between real mean thresholds (blue line) and model predictions (red dashed line) for the mice on the left based on a regressor model trained on ABR waveforms from 1-3 months of age (see **Fig 8**).

Model	Recall (%)	Sensitivity (%)	Precision (%)	ROC AUC (%)	Balanced accuracy (%)	F1 Score (%)
**HC**	82.57 ± 15.91	90.17 ± 12.36	89.38 ± 12.82	91.95 ± 9.86	86.37 ± 10.49	84.84 ± 12.08
**MLP**	78.71 ± 14.70	89.72 ± 11.53	88.18 ± 13.25	93.90 ± 7.14	84.22 ± 10.25	82.35 ± 12.15
**RF**	75.00 ± 14.02	86.83 ± 12.69	84.81 ± 13.60	89.61 ± 8.63	80.92 ± 10.05	78.77 ± 11.19
**Rckt**	84.79 ± 13.79	88.78 ± 11.80	88.30 ± 10.84	86.78 ± 8.45	86.78 ± 8.45	85.56 ± 9.40
**SVM**	85.86 ± 11.96	83.83 ± 15.45	84.35 ± 13.69	93.68 ± 8.41	84.85 ± 10.40	84.35 ± 10.29
**XGB**	73.43 ± 16.73	84.83 ± 13.42	82.79 ± 13.98	88.02 ± 10.60	79.13 ± 10.68	76.71 ± 12.22

S1 Table: Cross validation results for "Global" models.

Scores are mean ± SD across 25 folds. HC: HiveCoteV2.0; MLP: multilayer perceptron. RF: random forest; Rckt: ROCKET; SVM: support vector classifier; XGB: XGBoost.

**Metric**	**Main effect (Friedman test)**	**Nemenyi posthoc test**
**Recall**	P<0.0001	SVM - RF: P=0.0420
		SVM - XGB: P=0.0300
		all other comparisons: n.s.
**Specificity**	n.s. (P=0.0724)	
**Precision**	n.s. (P=0.0844)	
**ROC AUC**	P<0.0001	HC - Rckt: P=0.0113
		HC - XGB: P=0.0469
		MLP-Rckt: P=0.0003
		MLP-XGB: P=0.0022
		RF-MLP: P=0.0300
		RF-SVM: P=0.0377
		Rckt-SVM: P=0.0005
		SVM-XGB: P=0.0029
		all other comparisons: n.s.

S2 Table: P-value summary table comparing cross validation metrics among different “Global” models.

Comparisons were made using the Friedman test followed by the Nemenyi post-hoc test (**Fig 3b-e**).

Model	Recall (%)	Sensitivity (%)	Precision (%)	ROC AUC (%)	Balanced accuracy (%)	F1 Score (%)
**HC**	83.43 ± 13.05	86.50 ± 13.12	85.70 ± 12.46	90.77 ± 7.25	84.96 ± 8.88	83.82 ± 10.32
**MLP**	81.64 ± 15.87	84.50 ± 16.74	84.16 ± 15.24	90.25 ± 8.91	83.07 ± 11.44	81.80 ± 12.99
**RF**	77.93 ± 16.46	80.00 ± 17.71	80.10 ± 15.96	90.92 ± 7.67	78.96 ± 11.93	77.64 ± 12.43
**Rckt**	82.29 ± 14.05	85.06 ± 16.01	84.80 ± 14.07	83.67 ± 9.91	83.67 ± 9.91	82.55 ± 11.22
**SVM**	83.36 ± 15.58	77.17 ± 20.35	78.47 ± 15.41	90.97 ± 8.75	80.26 ± 12.80	79.82 ± 12.96
**XGB**	80.71 ± 16.17	77.56 ± 19.28	78.50 ± 15.15	88.49 ± 10.34	79.13 ± 12.42	78.41 ± 12.43

S3 Table: Cross validation results for "Click" models.

Scores are mean ± SD across 25 folds. HC: HiveCoteV2.0; MLP: multilayer perceptron. RF: random forest; Rckt: ROCKET; SVM: support vector classifier; XGB: XGBoost.

Metric	Main effect (Friedman test)	Nemenyi posthoc test
**Recall**	n.s. (P=0.1007)	
**Specificity**	P=0.0026	HC-SVM: P=0.0420
		all other comparisons: n.s.
**Precision**	P=0.0166	all pairwise comparisons: n.s.
**ROC AUC**	P=0.0004	Rckt-HC: P=0.0059
		Rckt-MLP: P=0.0420
		Rckt-RF: P=0.0268
		Rckt-SVM: P=0.0004
		all other comparisons: n.s.

S4 Table: P-value summary table comparing cross validation metrics among different "Click" models.

Comparisons were made using the Friedman test followed by the Nemenyi post-hoc test (**Fig 5b-e**)

Model	Cohort	Recall (%)	Sensitivity (%)	Precision (%)	ROC AUC (%)	Balanced accuracy (%)	F1 Score (%)
HC	Primary	79.21 ± 15.08	89.33 ± 10.11	87.42 ± 11.99	92.65 ± 6.88	84.27 ± 9.42	82.31 ± 11.35
	Replication	80.31 ± 10.48	87.75 ± 10.90	85.10 ± 11.25	91.95 ± 5.31	84.03 ± 6.33	81.82 ± 7.27
	Combined	76.51 ± 9.97	86.45 ± 8.13	83.16 ± 8.61	89.85 ± 4.91	81.48 ± 5.04	79.01 ± 5.90
MLP	Primary	78.86 ± 18.19	84.44 ± 16.34	83.43 ± 15.75	88.46 ± 9.92	81.65 ± 12.59	79.82 ± 14.64
	Replication	76.36 ± 12.92	90.75 ± 7.68	87.18 ± 9.41	91.58 ± 5.04	83.55 ± 7.04	80.68 ± 8.64
	Combined	73.89 ± 9.10	88.39 ± 7.34	84.47 ± 8.58	89.16 ± 4.79	81.14 ± 5.94	78.45 ± 7.08
RF	Primary	79.50 ± 16.22	79.17 ± 14.53	78.58 ± 13.91	89.83 ± 6.84	79.33 ± 11.08	78.01 ± 12.28
	Replication	71.51 ± 12.61	85.50 ± 7.38	79.58 ± 8.73	86.42 ± 6.50	78.51 ± 6.96	74.69 ± 8.59
	Combined	66.57 ± 10.25	80.16 ± 11.43	74.73 ± 11.22	82.98 ± 5.49	73.36 ± 6.40	69.60 ± 7.21
Rckt	Primary	77.50 ± 17.17	89.78 ± 11.96	88.10 ± 13.39	83.64 ± 10.73	83.64 ± 10.73	81.38 ± 13.11
	Replication	81.23 ± 13.57	89.75 ± 8.82	86.81 ± 9.71	85.49 ± 7.85	85.49 ± 7.85	83.13 ± 9.48
	Combined	77.37 ± 8.22	87.28 ± 7.25	83.86 ± 8.02	82.32 ± 4.91	82.32 ± 4.91	80.06 ± 5.64
SVM	Primary	83.36 ± 14.59	77.22 ± 20.31	78.57 ± 15.12	91.00 ± 8.61	80.29 ± 12.40	79.92 ± 12.30
	Replication	84.21 ± 10.77	86.50 ± 10.93	84.14 ± 11.91	93.55 ± 4.58	85.35 ± 7.12	83.43 ± 8.15
	Combined	85.07 ± 6.99	85.42 ± 9.99	83.70 ± 8.68	92.26 ± 3.66	85.25 ± 5.08	83.94 ± 5.17
XGB	Primary	78.36 ± 17.03	82.00 ± 14.95	80.79 ± 14.45	86.89 ± 9.35	80.18 ± 11.61	78.52 ± 13.01
	Replication	75.74 ± 10.62	87.00 ± 6.49	81.95 ± 7.83	89.65 ± 5.55	81.37 ± 6.64	78.38 ± 7.80
	Combined	67.81 ± 10.48	80.84 ± 10.93	75.49 ± 10.95	84.60 ± 5.28	74.33 ± 6.56	70.71 ± 7.64

S5 Table: **Cross validation results for "Click" models for the primary and replication cohorts.**

Scores are mean ± SD across 25 folds. HC: HiveCoteV2.0; MLP: multilayer perceptron. RF: random forest; Rckt: ROCKET; SVM: support vector classifier; XGB: XGBoost.

Model	Hyperparameter	Range	Value	Description
Random Forest classifier	n_estimators	50–1000	100	Number of trees in the forest
	max_depth	None, 5–50	None	Maximum depth of each tree
	min_samples_split	2–10	5	Minimum samples required to split an internal node
	min_samples_leaf	1–8	1	Minimum samples required at each leaf node
	bootstrap	[True, False]	True	Whether to use bootstrap samples for building trees
	max_features	[‘auto’, ‘sqrt’, ‘log2’]	‘sqrt’	Number of features considered for the best split
	class_weight	[None, ‘balanced’]	None	Weight adjustments to address class imbalance
SVM classifier	C	0.01–1	0.01	Regularization parameter
	kernel	[‘linear’, ‘rbf’, ‘poly’, ‘sigmoid’]	‘poly’	Kernel type
	gamma	[‘scale’, ‘auto’, 0.001–0.1]	0.01	Kernel coefficient for certain kernels
	degree	2–4	2	Degree of polynomial kernel
	shrinking	[True, False]	True	Whether to use shrinking heuristic
	class_weight	[None, ‘balanced’]	None	Adjusts weights for handling class imbalance
XGBoost classifier	n_estimators	50–200	200	Number of boosting rounds
	learning_rate	0.01–0.1	0.1	Boosting learning rate
	max_depth	3–10	3	Maximum tree depth for base learners
	subsample	0.6–1.0	0.6	Fraction of samples used per boosting round
	colsample_bytree	0.6–1.0	0.8	Fraction of features used per tree
MLP classifier	hidden_layer_sizes	[(50,), (100,), (150,), (100, 50)]	(150,)	Number and size of hidden layers
	activation	[‘relu’, ‘tanh’]	‘tanh’	Activation function for hidden layers
	learning_rate_init	0.001–0.1	0.001	Initial learning rate
	alpha	0.001–0.1	0.05	L2 regularization term to prevent overfitting
	max_iter	100–300	100	Maximum number of training iterations
ROCKET classifier	num_kernels	100–5000	5000	Number of random convolution kernels
	max_dilations_per_kernel	16–64	16	Maximum dilations per kernel
	n_features_per_kernel	2–8	2	Number of features per kernel
	use_multivariate	[‘yes’, ‘no’]	‘yes’	Use multivariate data
Random forest regressor - threshold	n_estimators	100-400	200	Number of trees
	min_samples_split	2-4	3	Minimum number of samples required to split an internal node
	min_samples_leaf	1-5	4	Minimum number of samples required to be at a leaf node
Random forest regressor	n_estimators	100-400	400	Number of trees
	min_samples_split	2-4	2	Minimum number of samples required to split an internal node

– Wave 1 amplitude	min_samples_leaf	1-5	2	Minimum number of samples required to be at a leaf node
Random forest regressor – Wave 1 latency	n_estimators	100-400	300	Number of trees
	min_samples_split	2-4	2	Minimum number of samples required to split an internal node
	min_samples_leaf	1-5	1	Minimum number of samples required to be at a leaf node
Global parameters	random_state		42	Initialisation of random seeds

S6 Table: Hyperparameters of the models.

For each hyperparameter, the search range used for optimization with 5-fold cross-validation and the final selected value are shown.
